# Transduction Mechanisms, Micro-Structuring Techniques, and Applications of Electronic Skin Pressure Sensors: A Review of Recent Advances

**DOI:** 10.3390/s20164407

**Published:** 2020-08-07

**Authors:** Andreia dos Santos, Elvira Fortunato, Rodrigo Martins, Hugo Águas, Rui Igreja

**Affiliations:** CENIMAT|i3N, Departamento de Ciência dos Materiais, Faculdade de Ciências e Tecnologia, Universidade Nova de Lisboa, Campus de Caparica, 2829-516 Caparica, Portugal; ass.santos@campus.fct.unl.pt (A.d.S.); emf@fct.unl.pt (E.F.); rm@uninova.pt (R.M.)

**Keywords:** electronic skin, pressure sensors, micro-structuring, health monitoring, functional prosthesis, robotics, human-machine-interfaces

## Abstract

Electronic skin (e-skin), which is an electronic surrogate of human skin, aims to recreate the multifunctionality of skin by using sensing units to detect multiple stimuli, while keeping key features of skin such as low thickness, stretchability, flexibility, and conformability. One of the most important stimuli to be detected is pressure due to its relevance in a plethora of applications, from health monitoring to functional prosthesis, robotics, and human-machine-interfaces (HMI). The performance of these e-skin pressure sensors is tailored, typically through micro-structuring techniques (such as photolithography, unconventional molds, incorporation of naturally micro-structured materials, laser engraving, amongst others) to achieve high sensitivities (commonly above 1 kPa^−1^), which is mostly relevant for health monitoring applications, or to extend the linearity of the behavior over a larger pressure range (from few Pa to 100 kPa), an important feature for functional prosthesis. Hence, this review intends to give a generalized view over the most relevant highlights in the development and micro-structuring of e-skin pressure sensors, while contributing to update the field with the most recent research. A special emphasis is devoted to the most employed pressure transduction mechanisms, namely capacitance, piezoelectricity, piezoresistivity, and triboelectricity, as well as to materials and novel techniques more recently explored to innovate the field and bring it a step closer to general adoption by society.

## 1. Introduction

Human skin is the largest organ of human body and has fascinating properties. It acts as a heat insulator to keep the body temperature constant, nonetheless also plays a crucial role in the cooling process of the body, for example, during intensive physical activity or when the external temperature is excessively high [1]. The skin protects the inner organs from external pathogenic agents, ultraviolet (UV) light, and multiple other aggressions, maintaining body homeostasis [1]. This organ is also an important interface with the surroundings due to thousands of biological receptors, scattered all over it, that are specialized in the detection of pressure, touch, vibration, tickle, heat, cold, and pain [2]. Furthermore, skin possesses sweat glands, essential structures for one of the cooling mechanisms of human body [1].

Amidst all the complexity of human skin, sensory ability has triggered scientific curiosity the most and instigated researchers to produce an electronic surrogate, the so called electronic skin (e-skin), also endowed with the perception of various external stimuli, such as mechanical stimuli, temperature, and humidity, while keeping or even surpassing the key qualities of human skin [3,4,5]:

- Low thickness—skin thickness (generally the sum of epidermis and dermis thicknesses) varies with the anatomic location, fluid content, age, and gender [6,7]. The breast skin can be as thick as approximately 6 mm (in males) [8], while the eyelid skin only reaches 800 µm [9];
- Stretchability—also variable with age, newborns skin can be subjected to a deformation of 75% before rupture, while for the elderly this value decreases to 60% [7];
- Flexibility—skin is highly flexible, especially in some anatomical places. For example, during squat position, the knee, and consequently the skin on it, can bend about 110° [10];
- Conformability—skin covers body tissues in a conformal way, following its exact shape, which allows the perception of movement of internal structures, namely blood vessels, muscles, and tendons, at its surface;
- Sweat induction—especially when the surroundings are warmer than skin temperature, sweat is the only mechanism for the body to lose heat [1]. Furthermore, sweat is an important mirror of health condition, given its high content in several metabolites that are closely related to health disorders, physical activities, and food consumption [11,12]. Therefore, it is highly desirable and useful to achieve an e-skin able to induce sweat, analyze these chemical molecules, and monitor, in real-time, the health status of an individual [12,13,14,15].

**Figure 1:**
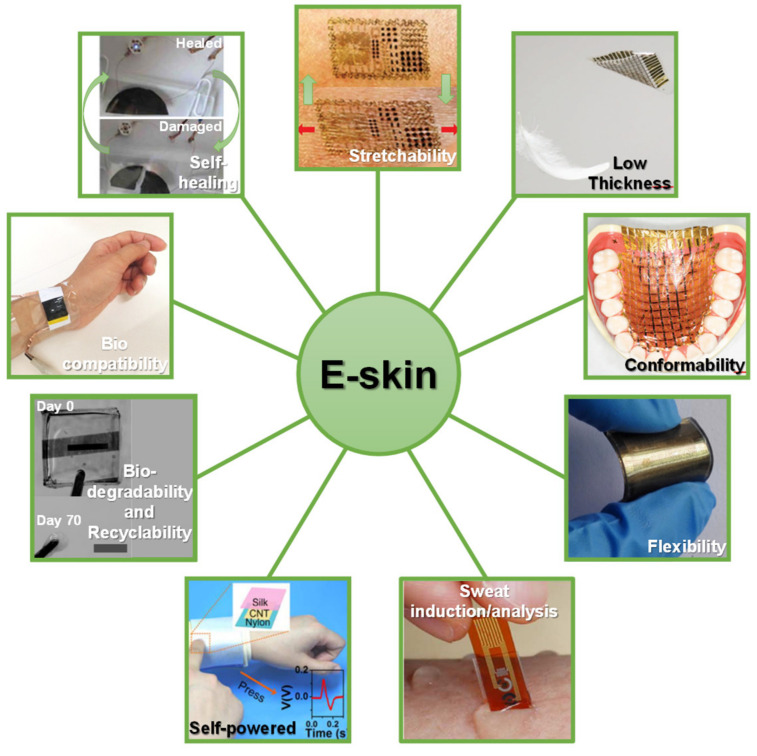
E-skin key features. Images adapted from: self-healing [16] (Copyright © 2020, Springer Nature), stretchability [17] (Copyright © 2020, The American Association for the Advancement of Science), low thickness and conformability [18] (Copyright © 2020, Springer Nature), flexibility [19] (© 2020 Acta Materialia Inc. Published by Elsevier Ltd. All rights reserved.), self-powered (reprinted with permission from [20]. Copyright 2018 American Chemical Society, Washington, WA, USA), biodegradability and recyclability [21] (Copyright © 2020 WILEY-VCH Verlag GmbH & Co. KGaA, Weinheim, Germany), biocompatibility [22] (Copyright © 2020 WILEY-VCH Verlag GmbH & Co. KGaA, Weinheim, Germany), sweat induction/analysis [14].

Other features such as biocompatibility, biodegradability and recyclability, self-healing, and self-powering have gained visibility to increase the potential of e-skin platforms [4]. With the purpose of making this technology widely available to the population in economic terms, it is highly important that e-skins may be produced in a low-cost way without compromising their functionality and efficiency. Therefore, envisioning a mass production of these devices to serve a large population, self-healing, biodegradability, and recyclability features get a particular importance as a contribution for a green economy. In fact, part of these e-skins may be intended for a disposable use or, due to their properties, may degrade quickly, which requires the repair or replacement of some components and a sustainable way to deal with the non-rectifiable parts [3]. The key features of an e-skin are illustrated in Figure 1.

### 1.1. Human Skin as Inspiration

**Figure 2:**
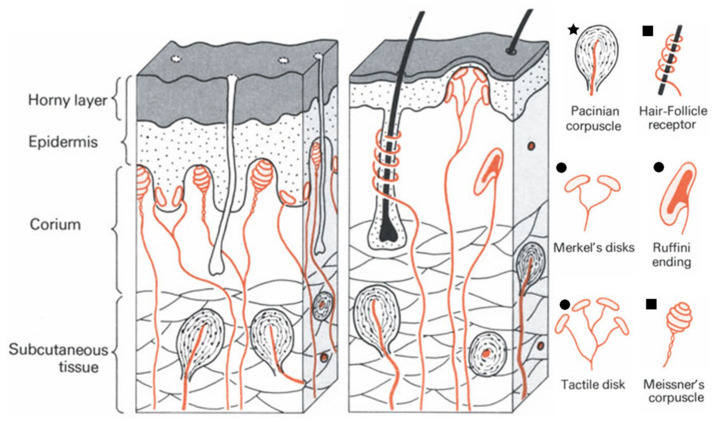
Types of mechanoreceptors present in hairless (**left**) and hairy (**right**) skin (adapted from [2], Copyright © 2020, Springer Verlag). The mechanoreceptors marked with 🟊, ⏹ or ⏺ are, respectively, very fast, moderately fast, or slow adapting.

The receptors of the human skin that detect the mechanical stimuli are called mechanoreceptors, which can be classified according to their adaptation to the stimulus [2]. Tactile disks, Ruffini endings, and Merkel’s disks adapt slowly to the stimulus, and so they are able to detect maintained stimuli like a constant pressure since they discharge stimulus-induced action potentials in a continued way [2]. These mechanoreceptors also provide information on the intensity of mechanical stimulus, which translates into skin indentation, as a function of the discharging frequency [2]. In fact, Merkel’s disks can be sensitive to small indentations of only 1 µm [23]. Meissner corpuscles and hair-follicle receptors have a moderately rapid adaptation, and so they are considered to be velocity detectors because they only discharge during movement, for example of a body hair, with a discharging rate that increases with the stimulus velocity [2]. Pacinian corpuscles adapt very rapidly and so they are considered as acceleration detectors [2]. Their discharging is related to the frequency of the stimulus (a vibration, in this case), within a certain frequency range, up to 700 Hz [2]. Figure 2 shows all the types of mechanoreceptors present in both hairless and hairy skin. Despite their differences, several types of mechanoreceptors may respond simultaneously to stimuli of different nature, which makes the distinction between pressure and touch a difficult endeavor [2].

Some mechanical stimuli are very subtle, such as a fly landing on the hand [24]; still, human skin is able to perceive those signals through a micro-structuring strategy. In fact, human skin is composed of three major layers: epidermis, dermis, and subcutaneous fat [25]. At its surface, it is possible to distinguish a series of tiny ridges and valleys that form the fingerprints, and in the interface of epidermis and dermis there is also a set of intermediate ridges which are aligned with the ridges of the fingerprints [25]. Some mechanoreceptors such as the slow adapting ones are found over the tip of these intermediate ridges (as illustrated in Figure 2), which does not seem to occur randomly [25]. Through simulations, it has been shown that these intermediate ridges focus and amplify the stresses at their tips, which allows the mechanoreceptors to respond to subtle stresses, thus increasing the sensitivity of skin [25]. This natural micro-structuring of skin inspired several works where e-skin pressure sensors with a greater pressure sensitivity were pursued, as will be described ahead.

### 1.2. Electronic Skin

**Figure 3:**
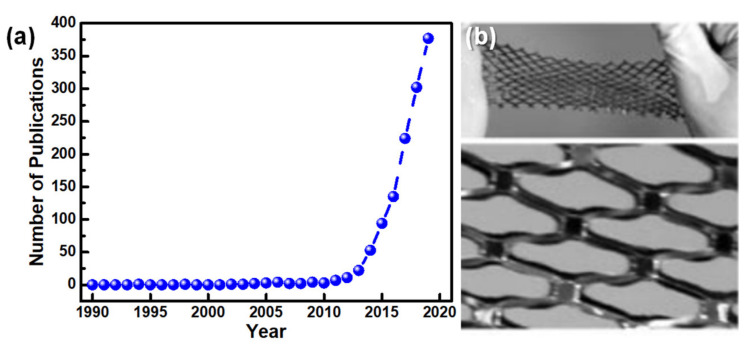
The beginning and growth of e-skin field. (**a**) Number of publications with the expression “electronic skin” on their content since 1990 (numbers estimated through Web of Science results). (**b**) E-skin developed by the group of Takayasu Sakurai in 2005 [29] [Copyright (2005) National Academy of Sciences, USA].

Research into e-skin essentially took off in the beginning of the 21st century, as Figure 3a illustrates, with the groups of Stephanie Lacour [26,27] and Takayasu Sakurai [28,29] giving pillar contributions to the field. The first group explored several strategies to reach stretchable conductors, such as the induction of spontaneous wrinkling by compressive stress that is built into a thin gold film evaporated on polydimethylsiloxane (PDMS), allowing a 22% stretching without losing the electrical properties of the conductive film [26], or depositing a conductive thin film over a pre-stretched PDMS substrate, so that both layers acquire a wavy pattern that can accommodate a large strain [27]. The second group developed organic transistors, with a mobility of 1 cm^2^/Vs (in the saturation regime) and an on/off ratio of 10^5^ to 10^6^, entirely produced out of soft materials (except for the electrodes) integrated with pressure sensors, aiming for the production of a flexible and conformable large area e-skin [28,29]. This e-skin could withstand a strain of 25% due to a net-shape structure, shown in Figure 3b, and did not display signs of significant output deterioration, even after 2000 cycles of 20% strain [29].

The massive interest in e-skin is highly motivated by the plethora of applications in which it may be employed, such as health monitoring [30,31,32,33,34], functional prosthesis [35,36,37], robotics [38,39,40], and human-machine-interfaces (HMI) [41,42,43]. Therefore, it is not surprising that the e-skin market value is estimated to be around USD 464.04 million in 2020, and is expected to reach USD 1,719.38 million by 2025 [44]. For these applications, pressure sensors play a crucial role. Many reviews have been published in the field over the last years. The first high impact review was performed by the group of Zhenan Bao in 2013 and had a broad coverage of many key features of e-skin devices [4]. The paper explored capacitive, piezoresistive, and piezoelectric tactile sensors, as well as chemical and biological sensors, with a small chapter concerning some applications of e-skin devices [4]. The encapsulation of devices was briefly referred, with respective advantages and disadvantages [4]. In 2015, another review exclusively focused on health monitoring applications (describing temperature, heartbeat, respiration, and glucose sensors, as well as drug delivery pumps, wireless systems, and displays) [3]. In 2015, Zhong Lin Wang and colleagues covered different pressure transduction mechanisms (except triboelectricity), strategies to improve the performance of the e-skin devices in terms of flexibility, stretchability, covered area, and resolution, and trends to make these devices self-powered, multifunctional, and able to transmit information through wireless technology [5]. Flexible pressure sensors and their main transduction mechanisms (except triboelectricity) were reviewed in another paper that also explored key characterization parameters, important applications, and relevant materials for substrates, active layers, and electrodes [45]. The article further focused on parameters that should be optimized and pertinent features [45]. In 2016, Zhenan Bao and co-workers reviewed strategies that are explored to pursue e-skin devices with mechanical properties similar to human skin, sensing properties (temperature, static force/pressure, strain, and dynamic force), and biomedical applications [46]. Self-powered e-skin sensors, piezoresistive sensors, and capacitive sensors were overviewed by Hyunhyub Ko and colleagues in 2016 [47]. The micro- and nano-structuring importance for sensor performance was briefly explored, as well as some applications [47]. From the same year, another paper reviewed flexible and/or stretchable sensors to monitor temperature, pressure (except triboelectric), and strain, as well as their integration with each other or other elements/sensors, and some health monitoring and functional prosthesis applications [48]. In 2018, one paper covered the fundamental components of smart wearables, namely flexible and/or stretchable substrates, stretchable conductors, active materials, wireless units, displays, and power supply units, with a varied range of applications [49], while another review covered the main types of flexible pressure sensors (except triboelectric), the materials employed in these devices, and relevant applications [50]. The main types of e-skin devices were overviewed in a paper from 2019, with a section dedicated to e-skins based on organic thin-film transistors [51]. Another review from the same year covered the main materials employed in several components of flexible sensors, as well as different types of sensors, namely strain, pressure (except triboelectric), temperature, humidity, magnetic, chemical, electromagnetic radiation, multimodal, electropotential, orientation, and ultrasonic [52]. The finite element analysis simulation was also addressed, as well as signal conditioning and processing circuits, several applications, and cases where the encapsulation of the devices was pursued [52]. The main type of sensors for flexible wearables, namely force (except triboelectric), temperature, physiological biochemical, and multifunctional, as well as nanogenerators, were focused in a work from 2019 [53]. Ni Zhao and co-workers reviewed the key figures-of-merit of pressure sensors, the main types of sensors and respective materials and some micro-structuring and fabrication techniques, with exploitation of sensors’ theoretical modelling and some applications [54]. A paper from 2019 covered several e-skin sensors, namely mechanical (including pressure and strain sensors), temperature, and humidity, as well as respective materials [55]. Some key features of these e-skin devices and micro-structuring techniques were approached [55]. Materials to pursue the key features of e-skin devices, as well as several types of sensors, such as pressure (except triboelectric), motion, temperature, humidity, biochemical, chemical, and multiplex, drug delivery systems, and printing technologies for the fabrication and micro-structuring of sensors were overviewed in another review from that year [56]. The group of Steve Park revised strategies to achieve stretchable devices, materials for self-healing and also biocompatible devices [57]. They explored pressure and strain sensors (with brief mentions to some micro-structuring techniques for performance improvement), temperature sensors, slip and force vector sensors, multifunctional sensors, chemical sensors, and electrophysiological sensors [57]. Approaches to achieve tactile mapping, large-area fabrication, wireless communication, neuromorphic devices, and electrodes for neural interfaces were also overviewed, as well as applications [57]. In 2020, a paper presented different pressure sensors (except triboelectric), some well-implemented micro-structuring techniques and resultant micro-structures [58]. Another paper reviewed materials for e-skin devices and different types of sensors (pressure, strain, temperature, and multifunctional), with a brief overview over self-powered units and array devices [59]. The group of Caofeng Pan explored piezoresistive pressure sensors, their key parameters, materials, and designs [60]. In 2020, Hyunhyub Ko and colleagues revised human skin and other natural systems as inspiration for e-skin devices, with brief mentions to some micro-structuring techniques and applications (monitoring of motion and health, and functional prosthesis) [61].

Overall, the majority of the published reviews cover not only pressure sensors but also other sensors and e-skin units, therefore, the description of pressure sensors is typically not very detailed [3,4,46,48,49,52,53,55,56,57,59]. Especially concerning the first reviews, triboelectric pressure sensors are not mentioned, possibly because their development is more recent, or the differences between the different types of pressure sensors are not clearly stated and explored [3,4,5,45,46,47,48,50,52,53,56,58,60]. Micro-structuring is a highly relevant topic in the field of e-skin pressure sensors, as it will be explored in the next sections, yet it is not emphasized in most works, or not broadly covered [3,4,5,45,46,47,48,49,50,51,52,53,54,56,57,58,59,60,61]. Some reviews are also not very extensive in the presentation of applications where the pressure sensors can be explored [3,4,5,46,51,53,54,55,56,58,59,60,61]. Finally, few reviews compiled the most relevant works in tables with key features of pressure sensors for an easy performance comparison, and regarding those that did, the table is not very extensive (not in works nor in details) [45,48,50,52,55,58]. Therefore, this review summarizes in one place comprehensive and vital aspects of pressure sensors, namely transduction mechanisms, micro-structuring techniques, most employed materials, and applications, that are typically not found together in other reviews. Regarding other types of interesting sensors and devices, such as strain sensors, humidity sensors, temperature sensors, biosensors, chemical sensors, gas sensors, antennas, energy harvesters, amongst others, many reviews are available for consulting [3,4,5,34,48,62,63,64,65,66,67,68,69,70,71].

## 2. Pressure Sensors

Pressure (force per unit area) is being constantly monitored in the human body. The blood pressure, which is the force exerted by blood per unit area of the vessel wall as a result of the periodic contraction and relaxation of the heart, is sensed by special nerve receptors called baroreceptors, located in the arterial wall [1]. Their feedback is sent to the brain to activate mechanisms for pressure increase or decrease when blood pressure is excessively low or high, respectively [1]. As explained in Section 1.1, the mechanoreceptors present in the skin are crucial to detect several mechanical stimuli, such as pressure, friction, torsion, vibration, bending, and stretching, allowing one to perceive touch, tickle, and other interactions of the surroundings with the body [2]. These and other receptors (namely proprioceptors) are also extremely important for the proprioception (notion of the body position, movement, and force) and the perception of body in space, allowing a precise control of body interactions with the surroundings, such as walking, objects grasping, and textures perception [2,4].

Given the relevance of pressure sensing in the human body, pressure sensors are similarly fundamental structures in an e-skin. In the context of robotics and prosthetics, these pressure sensors aim at the discrimination of normal and shear forces, tensile strain, and vibrations, so that the sense of grasping objects and manipulation, feeling textures, or proprioception may be simulated [4]. For health monitoring, pressure sensors may be used to monitor the blood pressure wave (BPW), heart rate, body movements, breathing, amongst others, which requires the sensors to achieve specific values of key parameters, given that some of the mentioned stimuli are very subtle [3,4,49]. These key parameters are used to characterize each sensor and constitute the figures of merit of pressure sensors: sensitivity, linearity, limit of detection (LOD), hysteresis, response and recovery or relaxation time, and stability [45,54]. A description of each parameter is given in Appendix A.

### 2.1. Pressure Transduction Mechanisms

The sense of pressure by e-skins is accomplished by transduction mechanisms that convert the pressure stimulus into an electrical signal, which can be measured by common electronic equipment [4]. Pressure sensors are then categorized according to the transduction mechanism they rely on for the pressure sensing, thus they can be majorly classified as capacitive, piezoelectric, piezoresistive, and triboelectric [4,54].

#### 2.1.1. Capacitive Sensors

A capacitive sensor is mainly composed of electrodes, a substrate, and an active layer that is commonly a dielectric material [54]. Under a pressure stimulus, this material suffers a deformation that leads to capacitance changes [54]. In a parallel plate capacitor, capacitance may be determined according to Equation (1) [45]:

(1) $$C=\frac{\varepsilon_{0}\varepsilon_{r}A}{d}$$

where *C* is the capacitance, *ε_0_* is the permittivity of vacuum, *ε_r_* is the relative permittivity of the dielectric material of the capacitor, *A* is the effective area of the electrode, and *d* is the distance between the two plates [45]. Given that *A* and *d* may be easily altered by external forces, Equation (1) can also be employed in capacitive sensors to monitor pressure changes or other stimuli [4]. The simplicity of the governing equation also simplifies the analysis of the sensors output, as well as their design [4]. In order to improve the performance of these sensors, the maximization of the dielectric material’s compressibility is pursued. This is frequently performed through the introduction of air-gaps [4], achieved by foamed dielectric materials [72,73] or through micro-structuring [24,74,75], and besides increasing the sensitivity of the sensor, they also contribute to the reduction in the sensor’s response and relaxation times, as well as hysteresis [24,76,77].

The miniaturization of these sensors must be performed with caution, since the size reduction of the sensor implies a reduction in its area, affecting its nominal capacitance and decreasing the signal-to-noise ratio or increasing the crosstalk between adjacent elements [30,43,72,74,75,76,78,79,80,81,82,83]. Another issue is related to the common use of polymers as dielectric layer. These materials have a high viscoelasticity, thus resulting in sensors with high response times and hysteresis [24,73].

**Figure 4:**
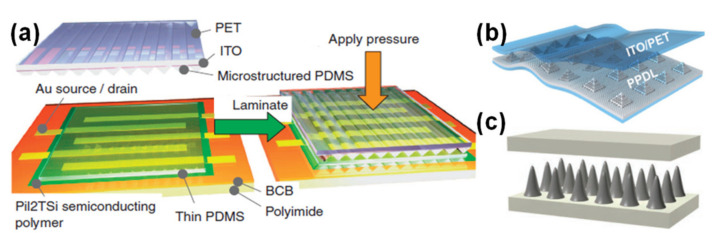
Capacitive e-skin sensors, developed by (**a**) Zhenan Bao and co-workers in 2013 [30] (Copyright © 2020, Springer Nature), (**b**) Steve Park and co-workers in 2019 (reprinted with permission from [85]. Copyright 2019 American Chemical Society, Washington, WA, USA), and (**c**) Run-Wei Li and co-workers in 2020 [86] (Copyright © 2020 WILEY-VCH Verlag GmbH & Co. KGaA, Weinheim, Germany).

Professor Zhenan Bao’s group has a vast experience in e-skin capacitive sensors. One of their first devices consisted of an organic field-effect transistor (OFET) where the dielectric component was a PDMS film with micro-pyramids, which contributed to the improvement of the pressure sensor by increasing its sensitivity while decreasing its relaxation time (comparatively to an unstructured dielectric) [24]. In 2011, this group created another type of sensor, meant to be highly flexible, stretchable, and transparent, which was based on electrodes of single-walled carbon nanotubes (SWCNTs) spray coated over a PDMS film and a dielectric layer of ecoflex [84]. Due to pre-strain cycles, the bundles of SWCNTs achieved a wavy pattern (in the strain direction) which was able to bear a certain level of strain with a reversible sheet resistance change [84]. In 2013, Zhenan Bao and colleagues took a step forward by creating an organic thin film transistor (OTFT) that was flexible, unlike his predecessor, and showed a greater sensitivity through the operation of the transistor in the subthreshold regime, where the source-drain current exhibited a superlinear dependency on the capacitance change induced by pressure [30]. In this regime, the sensor, shown in Figure 4a, could distinguish the BPW at the wrist with a high resolution [30].

To obtain astrain-insensitive matrix of capacitive sensors, the group of Steve Park explored the strategy of harder sensitive islands, made of PDMS, embedded in a softer elastomeric substrate of ecoflex, with an elastic modulus almost 40 times smaller than that of PDMS [85]. The dielectric layer was a set of porous PDMS micro-pyramids, whose holes were produced by sacrificial polystyrene spheres, as Figure 4b shows [85]. When the matrix was subjected to a strain of 50%, ecoflex concentrated a strain of 105.7%, while the PDMS islands only suffered a strain of 5.2% [85].

Through an innovative approach to micro-structure PDMS based on the mixing of the polymer with magnetic particles (silver coated with nickel) and subjecting it to a strong magnetic field to induce the formation of micro-needles as Figure 4c presents, the group of Run-Wei Li created a low-cost capacitive e-skin, whose micro-needles features could be fairly controlled through the concentration of magnetic particles or magnetic field intensity [86]. With heights ranging from 275 µm to 856 µm, and diameters ranging from 166 µm to 420 µm, the optimized micro-needles conferred the e-skin a modest sensitivity of 0.159 kPa^−1^ bellow 1 kPa [86].

#### 2.1.2. Piezoelectric Sensors

Piezoelectric sensors rely on piezoelectricity to transduce a pressure into an electrical signal. These sensors typically present a fast response time and high sensitivity, establishing them as suitable candidates to detect dynamic pressures, as vibrations and slip [87,88,89], yet inadequate to sense static pressures [88]. Given the pyroelectricity of piezoelectric materials, there is also the possibility of misinterpreting the output of a piezoelectric sensor in the presence of temperature changes [4,88,89].

The most popular piezoelectric materials used in e-skin sensors, energy harvesters, and other types of sensors are lead zirconate titanate (PZT) [89,90,91], zinc oxide (ZnO) [68,87,92,93,94,95,96,97,98,99,100,101], barium titanate (BaTiO_3_) [102,103], poly(vinylidene difluoride) (PVDF) and its co-polymers like poly[(vinylidenefluoride-co-trifluoroethylene] [P(VDF-TrFE)] and poly(vinylidene fluoride-co-hexafluoropropene) [P(VDF-HFP)] [88,99,102,103,104,105,106,107,108], and their piezoelectric coefficient, d_33_, can be found in Table 1. The relation between the d_33_ and the output of a piezoelectric sensor can be established by Equation (2):

(2) $$V=\frac{d_{33}\times F}{C}$$

where *V* is the voltage produced by the sensor, *C* is the capacitance, and *F* is the force applied to the sensor [109].

PZT and ZnO are commonly integrated into sensors in the form of thin films or nano/micro-structures to minimize their brittle nature [54,87,89,91,97,100]. PVDF polymers are highly flexible, yet require a poling process to maximize their typically low d_33_ constant [54,99,102,103,105,106,107,108]. The electrospinning of PVDF polymers fibers seems to eliminate the need of poling given that the process itself polarizes the polymer [104].

The pioneer work of Zhong Lin Wang’s group on ZnO nanostructures and their use in energy harvesting and pressure sensing left an important mark in the field of piezoelectric e-skins. Since their publication regarding the use of aligned ZnO nanowires to harvest energy from a mechanical stimulation with an atomic force microscope (AFM) tip [114], and posterior fabrication of a field effect transistor using a single ZnO nanowire to control the source to drain current through its bending, dependent on the applied pressure [95], there was an accentuated increase in publications focusing on piezoelectric systems, many of them coming from this group [87,97,99,114,115,116,117,118,119,120].

**Figure 5:**
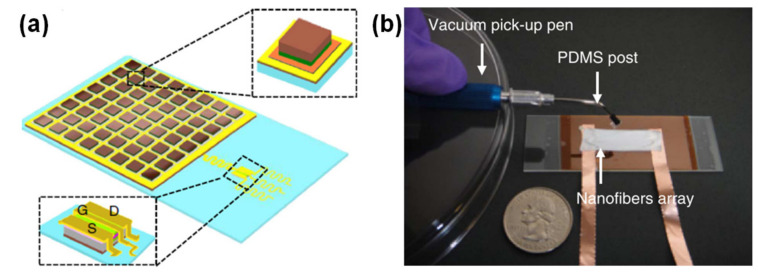
Piezoelectric e-skin sensors, developed by (**a**) John Rogers and co-workers in 2014 [90] (Copyright © 2020, Springer Nature) and (**b**) John Rogers and co-workers in 2013 [104] (Copyright © 2020, Springer Nature).

The group of John Rogers has worked on several piezoelectric pressure sensors, namely one based on several squares of thin PZT films connected to a silicon metal oxide semiconductor field effect transistor (MOSFET) through serpentine paths, to accommodate stretching until 30% [90], as Figure 5a illustrates. The role of the MOSFET was to amplify the voltage generated by the PZT elements upon applying pressure [90]. Due to the design of the sensor, the PZT elements were located in the neutral mechanical plane, in order to minimize the bending interference in the pressure measurement and allow the sensor to be used in curvilinear surfaces, such as the wrist, for the detection of the BPW [90]. Another highly bendable piezoelectric sensor was made of electrospun P(VDF-TrFE) nanofibers [104]. The mesh of P(VDF-TrFE) did not require a poling process, as the electrospinning of the fibers enhanced the orientation of piezoelectric active dipoles in the direction perpendicular to the longitudinal axis of the fibers [104]. Figure 5b shows a photograph of this sensor.

#### 2.1.3. Piezoresistive Sensors

Piezoresistivity is described as the effect of resistance change with an external mechanical stimulus [121]. With the piezoresistive effect, not only the geometrical parameters of an electrical conductor resistance are susceptible to change, but also the material resistivity itself in the case of some particular materials such as crystal semiconductors [121].

In piezoresistive sensors, the resistance change may rely on distinct mechanisms:

(i) Resistivity variations—in a semiconductor, as a result of band structure changes induced by pressure [122], or in composites, as a result of interparticle distance change [42,123,124]. For these cases, the following equation may be applicable (Equation (3)):
  (3) $$R=\frac{\rho \times l}{A}$$
  where *R* is the resistance, *ρ* is the material resistivity, *l* is the conductor length, and *A* is the transverse section area [121];
(ii) Contact resistance variations—through the modification of the geometry of the sensing element [19,31,125,126,127,128,129,130], by contact area changes induced in interlocked designs [38,87,131,132,133,134,135,136], or through contact area changes in foamy or spongy materials [41,137,138]. For these cases, the contact resistance is governed by Equation (4):
  (4) $$R_{C}\propto F^{-\frac{1}{2}}$$
  where *R_C_* is the contact resistance and *F* is the force [4]. This equation shows that sensors playing on contact resistance have a high sensitivity for low forces and a large working range [4] which can also be easily tuned through micro-structuring [87,127,132,139].

Piezoresistive sensors have a very simple design and readout mechanism [4,5,45]; however, their high hysteresis and long relaxation times, which are a consequence of the use of polymeric and viscoelastic materials in the sensors, require micro-structuring of those polymeric films, a strategy that is also employed in other types of pressure sensors and even energy harvesters [24,47,54,73,133,140,141,142]. Besides improving the parameters previously mentioned, the micro-structuring also enhances the sensitivity of the sensors, and it can be achieved through several strategies [54], explored ahead.

**Figure 6:**
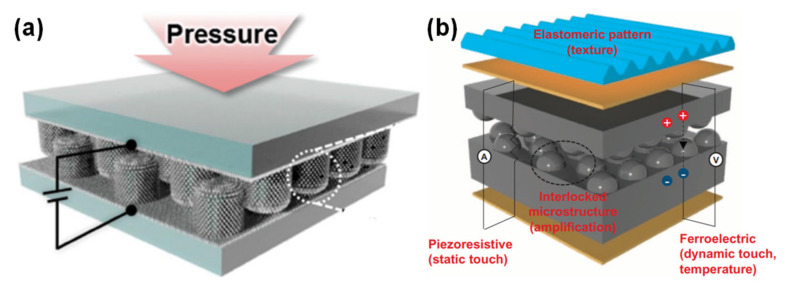
Piezoresistive and piezoelectric e-skin sensors developed by Hyunhyub Ko and co-workers in (**a**) 2015 [87] (Copyright © 2020 WILEY-VCH Verlag GmbH & Co. KGaA, Weinheim, Germany) and (**b**) 2015 [88].

Human skin structure has been an inspiration for several e-skins developed in 2015 by the group of Hyunhyub Ko [87,88], as illustrated in Figure 6. The group mainly explored interlocked geometries, mimicking the epidermal-dermal micro-ridges of human skin, in a piezoresistive [132,133,136] or piezoresistive and piezoelectric [87,88] configuration. In the piezoresistive e-skins, the interlocking strategy enabled an initial contact resistance defined by the contact points between the micro-pillars [132,136], micro-pyramids [136], or micro-domes [133,136] of a composite of PDMS and multiwalled carbon nanotubes (MWCNTs). Upon applying pressure, these micro-structures suffered severe deformation, which greatly increased the contact between them, thus decreasing the resistance of the device, mainly dominated by the tunneling resistance [132,133,136]. This operation mode allowed for high sensitivity values which could be tuned by adjusting the pitch or the size of the micro-structures [132]. For one of the hybrid configurations, PDMS micropillars were covered with ZnO nanowires and coated with a metallic layer to make them conductive and rigid, with the purpose of significantly increasing the contact area variations with pressure and assuring a fast response time with minimized hysteresis [87]. By removing the metallic coating from one of the micro-structured films, the e-skin could be used in a piezoelectric mode to detect mechanical vibrations of high frequency (up to 250 Hz) due to the enhanced bending of the ZnO nanowires induced by the interlocking geometry [87]. The other hybrid e-skin of the group could detect both temperature and pressure changes due to the piezoresistive and pyroelectric properties of the micro-dome structured composite of PVDF and reduced graphene oxide (rGO) [88]. This interlocked e-skin could further monitor the BPW at the wrist and the temperature simultaneously, while its piezoelectric performance allowed the detection of dynamic touch and acoustic waves [88]. Recently, in 2018, the group explored the impact of the micro-structures’ shape in the sensitivity of interlocked piezoresistive devices for the detection of different forces, while maintaining the size and pitch for all geometries [136]. These devices showed a much improved output in comparison with single micro-structured film devices, since the insulating layer is larger in the interlocked geometry, which increases the tunneling resistance, resulting in a lower current in the absence of pressure [136]. Even though the devices with pyramidal micro-structures presented a greater height compression with pressure, those with dome micro-structures exhibited a greater variation in contact area with pressure, leading to an extremely high sensitivity of 47 × 10^3^ kPa^−1^ [136].

Regarding the research conducted to avoid photolithography techniques for micro-structuring, the group of Seimei Shiratori actually developed a peculiar technique in which uncured PDMS would be slowly dropped over water [143]. Due to differences in density and surface tension, the uncured PDMS would float on water, and after some time, form a thin cured film [143]. Possibly due to temperature differences between air and water during curing, and succeeding post-curing bending cycles, fish-scale like structures arose on the surface of the PDMS film, on the side that was in contact with air [143]. This micro-structured film was then covered with poly(3,4-ethylenedioxythiophene)–poly(styrenesulfonate) (PEDOT:PSS) and graphene nanosheets to become conductive, and the pressure sensor was produced through the assembly of two films [143]. This piezoresistive sensor decreased its resistance with pressure and presented a high sensitivity of –70.86 kPa^−1^ under 1 kPa, which was enough to detect the BPW at the wrist [143]. Three-dimensional (3D) printing was also explored by Zhengchun Peng and co-workers [144]. The group developed an ink of thermoplastic urethane polymer (TPU), sodium chloride, and carbon black nanoparticles, and after printing it over PDMS, the sensing layer was submerged in water to remove the sodium chloride, subsequently forming a porous matrix [144]. Owing to the presence of several pores sizes (nanometer pores originated by the mixing of carbon black particles with TPU, tens of micrometers pores originated by the dissolution of sodium chloride, and larger pores originated by the 3D construction), the sensor presented three distinct sensitivities in a large pressure range that extended up to 800 kPa [144]. Although not being the first group to explore sandpaper as a mold for micro-structuring, Dawen Zeng and colleagues conducted a thorough research on the impact of different sandpapers in the sensitivity and pressure range of the respective piezoresistive sensors [130]. By using sandpaper that produced higher (250 µm on average) and larger micro-structures that were further covered with rGO, the sensors could achieve a sensitivity of 2.5 kPa^−1^ between 0 Pa and 1 kPa, 12 kPa^−1^ between 1 kPa and 50 kPa, 1051 kPa^−1^ between 50 kPa and 200 kPa, and 470 kPa^−1^ between 200 kPa and 400 kPa [130]. The excellent sensitivity values were due to the presence of micro-structures with different heights—the higher micro-structures would contact with the electrodes for smaller pressures, while the smaller micro-structures would contact with the electrodes for larger pressures [130]. Being larger, these micro-structures also required more pressure to deform and contact the electrode, therefore sandpaper with micro-structures of an average height of 15 µm presented a larger sensitivity of 600 kPa^−1^ between 1 kPa and 50 kPa. However, the structures’ deformation saturated more rapidly and so the sensitivity between 200 kPa and 400 kPa was smaller (5.3 kPa^−1^) [130].

#### 2.1.4. Triboelectric Sensors

**Figure 7:**
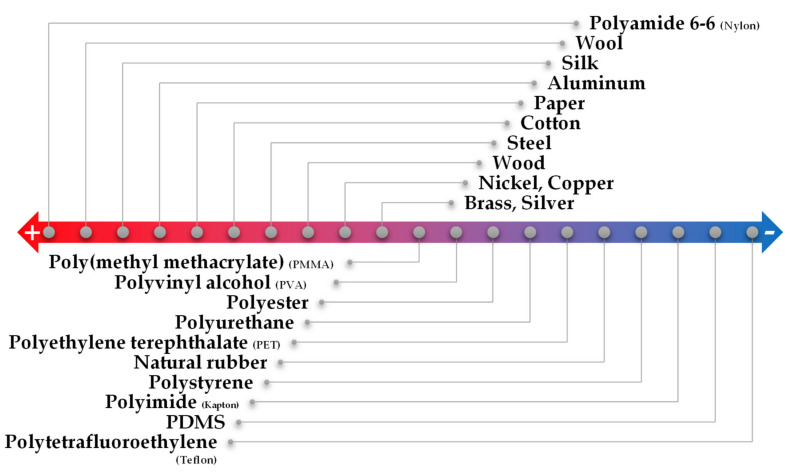
Triboelectric series for common materials following a tendency of easily losing electrons (+) to gaining electrons (−) (adapted from [146]).

Triboelectricity is the result of the combination of triboelectrification, which is the generation of charges at the surface of two different materials when they are rubbed, and electrostatic induction, a phenomenon of electricity-generation that is characterized by the flow of electrons between two electrodes, through an external load, to balance their potential difference [145]. This contact electrification phenomenon is potentiated by the difference of triboelectric polarity of each material: the higher the difference, the greater the amount of charges generated [47,146]. The tendency of some materials to lose or gain electrons when rubbed is commonly represented by the triboelectric series, presented in Figure 7. When the two materials are rubbed, opposite charges are induced at their surface, and after separation of the two materials, the respective electrodes generate compensating charges for each material so that the electrostatic equilibrium may be maintained [47]. An external circuit connected to both electrodes allows the electron flow between them, which can be amplified by increasing the contact area of the materials and by having a high separation-distance change [47]. In this class of sensors, it is also common to explore the micro-structuring of the materials to increase the friction area and, therefore, the triboelectrification [146]. When mixing nanoparticles in a polymeric matrix, the composite acquires a different permittivity and surface electrification if compared to the bare polymer, thus changing its triboelectric potential [146].

**Figure 8:**
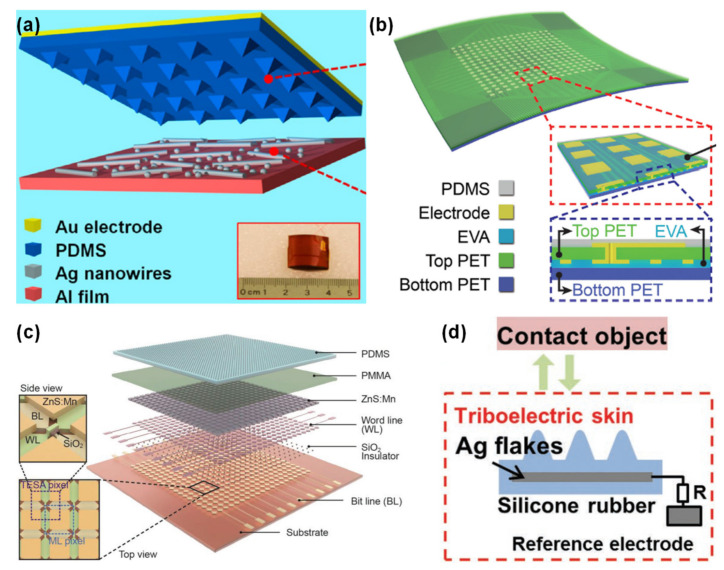
Triboelectric e-skin sensors, developed by Zhong Lin Wang and co-workers in (**a**) 2013 (reprinted with permission from [150]. Copyright 2013 American Chemical Society, Washington, WA, USA), (**b**) 2016 [153] (Copyright © 2020 WILEY-VCH Verlag GmbH & Co. KGaA, Weinheim, Germany), (**c**) 2017 [154] (Copyright © 2020 WILEY-VCH Verlag GmbH & Co. KGaA, Weinheim, Germany), and (**d**) 2018 [40] (Copyright © 2020 WILEY-VCH Verlag GmbH & Co. KGaA, Weinheim, Germany).

Despite their obvious capability to transduce mechanical stimuli into induced currents, this type of sensors are more suitable to detect dynamic pressures such as vibrations because their output is affected by the magnitude and frequency of the stimulus [20,54,147,148]. Amongst them, a great portion was developed by Zhong Lin Wang and co-workers, with emphasis on micro-structured films to increase the friction area of the sensors and enhance the triboelectric effect [40,148,149,150,151,152,153,154]. Figure 8 presents some of these triboelectric sensors to illustrate their design and materials.

### 2.2. Comparison of Transduction Mechanisms

Table 2 compares all the transduction mechanisms previously referred. One may conclude that different applications require different transduction mechanisms. For static pressure detection, capacitive and piezoresistive pressures are more suitable, since their output is maintained with the pressure stimulus, even though these sensors need a power supply to work. For dynamic and high frequency stimuli, piezoelectric and triboelectric sensors are more appropriate and have the advantage of being self-powered. For example, to monitor the BPW at the wrist, which is a low frequency signal (its monitoring typically requires a bandwidth of 20 Hz in the hypertension context, although it may extend to 200 Hz in other health issues such as heart failure [155]), and should not be affected by high frequency stimuli such as vibrations, the most suitable types of sensors are capacitive and piezoresistive.

### 2.3. Micro-Structuring Techniques and Materials

**Figure 9:**
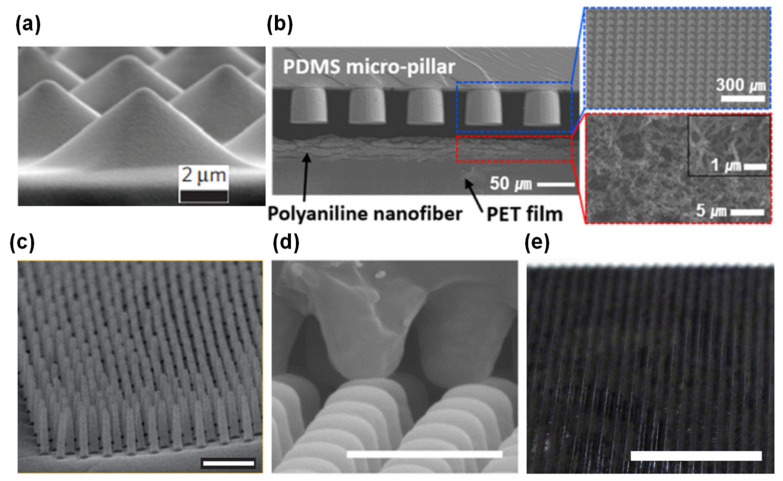
Micro-structures produced through photolithography techniques. (**a**) Pyramids [24] (Copyright © 2020, Springer Nature). (**b**) Pillars (reprinted with permission from [31]. Copyright 2015 American Chemical Society, Washington, WA, USA). (**c**) Hairs [131] (Copyright © 2020, Springer Nature). (**d**) Domes (reprinted with permission from [133]. Copyright 2014 American Chemical Society, Washington, WA, USA). (**e**) Triangular lines [41] (Copyright © 2020, Springer Nature).

The micro-structuring of the films composing the e-skin pressure sensors is a common strategy to improve their sensitivity while reducing their response and relaxation times, and hysteresis [24,54,76,77,146], being explored for all types of pressure sensors. The strategies most employed in the films micro-structuring (and respective materials) are the following:

- **Photolithography techniques to etch silicon wafers and produce molds.** Despite being expensive, complex, and time-consuming, this micro-structuring strategy is widely used to obtain highly regular and homogeneous patterns (shown in Figure 9) based on pyramids [19,24,125,126,129,136,156,157,158,159,160,161,162,163], pillars [31,87,132,136,159,164], hairs [131,165], domes (or semi-spheres) [133,136,139], triangular lines [30,41,160], and cubes [24]. These micro-structured films are typically made of PDMS [19,24,30,31,87,125,126,129,139,156,157,158,160,161,162,164,165] or composites of PDMS with MWCNTs [132,133,136]. For the case of piezoresistive sensors, PDMS micro-structures are commonly covered by SWCNTs or MWCNTs, deposited through spray-coating either directly on the PDMS [19,129] or previously on the mold before the PDMS deposition [156,158]. Metals deposited by vapor deposition methods such as gold [31,157], platinum [87,131], and nickel [87] have also been explored. Inkjet printing directly on PDMS is a more recent strategy to cover the films with MWCNTs [162] or a composite of PEDOT:PSS, polyurethane dispersion, and silver nanoparticles [161]. For the cases where only one film is micro-structured or an additional support is needed, substrates of polyethylene terephthalate (PET) [19,31,139,160], PET with indium tin oxide (ITO) [24,30,41,126,129], polyimide [30,161], PDMS [131,164], polyethylene [156], and polyethylene naphthalene (PEN) [165] have been employed.
- **Use of everyday objects as unconventional molds.** This approach is much less expensive than photolithography techniques, nevertheless it does not allow for design changes in the micro-structuring due to limitations regarding the objects available to act as molds. Several objects have been used as molds, from sandpaper [130,166,167,168,169,170,171] to paper [39], leaves of several plants’ species [38,43,142,172,173,174,175,176,177], insect wings [142], animals skin [178], and fabrics [140,179,180,181,182]. PDMS is once again the most chosen material for the micro-structured films [38,39,130,140,142,167,168,169,172,173,174,175,176,177,178,179,182], as well as PDMS-based composites with graphite [166,170] or carbon nanotubes (CNTs) [181]. For some sensors, it is common to coat PDMS films with gold [38,169], silver nanowires [39,173,176], rGO [130,168], CNTs [167,172], SWCNTs [179], graphene [172,174,175,182], and PEDOT:PSS [178] through vapor deposition methods [38,169], spray-coating [39,167,175,176], dip-coating [168,179], and transfer methods [172,174,182]. Polyimide [39,43,171] and PET with ITO [166,170,177] are common substrates as well.
- **Treatments of the sensing film**, such as PDMS heating [183,184], PDMS stretching and UV or oxygen plasma exposure [75,141,185,186], and self-assembly or chemical reaction [86,99,135,184,187,188,189,190,191]. Regarding the latter approach, the most common materials employed to achieve a certain level of micro-structuring are ZnO in several shapes [99,135,188,190], graphene [189], and silver particles [191]. For all strategies, the resultant micro-structuring has a limited level of tailoring.
- **Incorporation** of sponges [192,193,194,195,196,197,198,199], foams [200,201,202,203,204,205,206,207], paper [208,209,210,211,212], and natural or synthetic fabrics (such as cotton, leather, silk, polyamide fabric, polyester fabric, polypropylene fabric, polyurethane fibers, and tissue paper) [32,123,134,213,214,215,216,217,218,219,220,221,222,223] that are afterwards chemically modified to become conductive, typically by carbonization [123,196,216] or by dip-coating with rGO [192,195,219,221], graphene [134,223], CNTs of different types [209,213,220], or silver nanowires [195,212,218,221,222].
- **Production of porous films** through freeze-drying [137,224,225,226,227,228,229,230] or using sacrificial templates made of sugar [138,231,232,233,234], salt [233,234,235,236,237], or polystyrene spheres [73,238,239,240]. The most explored materials in these techniques are PDMS [73,138,231,232,234,235,237,238,240], graphene oxide [224,225,226], and ecoflex [233,236]. Despite their low-cost, all these techniques also have a limited level of design tailoring.
- **Fabrication of 3D** printed molds [78,241,242,243,244] (majorly to micro-structure PDMS or PDMS composites) or direct printing of materials with a 3D printer [245,246], which is a low-cost approach to achieve a micro-structuring, nonetheless typically only allows the achievement of structures with a size in the order of few mm due to printer and filament constraints.
- **Production of molds through laser engraving technique**. This is a quite recent strategy to avoid the high costs of common photolithography processes without losing the high customization degree of the micro-structuring design as it happens with the use of unconventional molds, presenting, therefore, a high benefit/cost ratio [22,109,247,248,249]. Essentially, the laser beam transfers a high amount of energy that induces the melting or degradation of a material, creating cavities whose shape can be controlled through the design imported to the equipment, laser power and speed, as well as the material itself [22,247]. The material with the cavities pattern can be posteriorly employed as a mold for soft lithography processes, commonly for micro-structuring PDMS [22,37,40,247,248,249,250,251,252,253] or PDMS composites [109]. For piezoresistive sensors, these films have then been coated with carbon ink (by spin-coating) [22,247,248,249], CNTs (by drop-casting) [37,250], silver nanowires (by spin-coating) [252], or rGO (by spray-coating) [253] to become functional.

**Figure 10:**
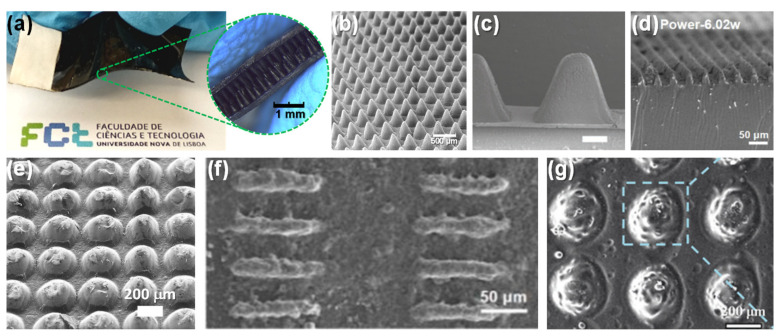
E-skins produced through laser engraving technique. (**a**) E-skin developed by Rui Igreja and co-workers in 2018 [22] (Copyright © 2020 WILEY-VCH Verlag GmbH & Co. KGaA, Weinheim, Germany). (**b**) SEM image of micro-cones of the e-skin in (a) [22] (Copyright © 2020 WILEY-VCH Verlag GmbH & Co. KGaA, Weinheim, Germany). (**c**) SEM image of triangular microprisms produced by Zhong Lin Wang and co-workers in 2018 (scale bar = 0.5 mm) [40] (Copyright © 2020 WILEY-VCH Verlag GmbH & Co. KGaA, Weinheim, Germany). (**d**) SEM image of triangular lines produced by Fuzhen Xuan and co-workers in 2018 [37] (© 2020 Elsevier B.V. All rights reserved). (**e**) SEM image of semi-spheres produced by Rui Igreja and co-workers in 2019 [247]. (**f**) SEM image of short micro-ridges produced by Fuzhen Xuan in 2019 [250] (Copyright © 2020, IOP Publishing). (**g**) SEM image of hemispherical microstructures produced by Tong Zhang in 2020 (used with permission of Royal Society of Chemistry, from [253]; permission conveyed through Copyright Clearance Center, Inc.).

The laser engraving technique for the micro-structuring of molds was introduced in the e-skin field by Rui Igreja and colleagues in 2018 [22]. The first e-skin developed by the group through this technique was based on interlocked micro-cones, covered with carbon coating, obtained through engraved acrylic molds [22]. Keeping the entire fabrication process based in low-cost strategies, the e-skin achieved a sensitivity of –2.52 kPa^−1^ bellow 160 Pa, a value comparable with sensors produced through expensive photolithography techniques or using unconventional molds, and suitable for the detection of the BPW at the wrist with great detail [22]. To prove the versatility of the technique, the group also explored the engraving of hard PDMS for the production of a piezoresistive sensor with interlocked micro-domes [247]. Possessing less compressible micro-structures, this e-skin could withstand large pressures with a fixed sensitivity of −6.4 × 10^−3^ kPa^−1^ (between 1.2 kPa and 100 kPa) [247]. This performance was more suitable for functional prosthesis or robotics, where it is relevant to have a stable sensitivity in a large pressure range, as it was verified when the e-skin was placed in a robotic arm to monitor the grasping and releasing of an object throughout 27,500 cycles without a degradation of the output [247]. Since 2018, other groups reported the use of this technique, either producing engraved molds for soft lithography processes [40,250,251,252,253], or directly engraving the polymeric films that integrate the sensor [37], achieving structures such as triangular microprisms [40], triangular lines [37], micro-ridges [250], micro-domes [250,252,253] and micro-cones [251]. Figure 10 illustrates some micro-structures achieved through the laser engraving technique.

The comparison of the most common approaches for the micro-structuring of films for pressure sensors (photolithography and unconventional molds) with the emerging strategy based on laser engraving is presented in Table 3.

### 2.4. State-of-the-Art

Since the pioneer work of the group of Takayasu Sakurai and the group of Stephanie Lacour, several e-skin pressure sensors have been reported, exploring the different transduction mechanisms previously mentioned and targeting distinct applications, as presented in Appendix B.

## 3. Applications

E-skin sensors have an enormous potential for diverse fields, and several of the platforms developed so far have been tested to testify those expectations, as presented in the next sections.

### 3.1. Health Monitoring

#### 3.1.1. Blood Pressure and Blood Pressure Wave

As described in Section 2, blood pressure (BP) is a vital signal defined as the force exerted by blood against any unit area of the vessel wall and due to the pulsatile nature of the heart’s blood pumping, BP alternates between systolic pressure and diastolic pressure, which occur due to the contraction or relaxation of the heart, respectively [1]. The systolic and diastolic BP values are considered to be in normal ranges when they are below 120 mm Hg and below 80 mm Hg, respectively [254]. If an adult presents systolic and diastolic BP values over 140 mm Hg and 90 mm Hg, respectively, then the subject is diagnosed with hypertension, meaning the BP values are excessively high [254].

The diagnosis of hypertension and its proper follow-up is highly relevant since this health issue is one of the most important risk factors for other serious and high burden diseases such as coronary heart disease, stroke, and renal failure [254,255]. Furthermore, high BP was the leading risk factor for global disease burden in 2010, ahead from high body-mass index or tobacco smoking, with 9.4 million of related deaths [256]. The global percentage of people diagnosed with hypertension is estimated to increase from 26.4% in 2000 to 29.2% in 2025 (about 1.56 billion adults affected) [257], which ultimately points towards the importance of an accurate hypertension diagnosis, a proper follow up of such patients, and adjusting the drug treatment or lifestyle with the help of continuous BP monitoring in the simplest and most convenient way for the patients. Given that the golden standards for non-invasive BP monitoring produce intermittent and uncomfortable measurements [254,258,259], the search has been stimulated for other non-invasive techniques able to continuously measure the BP, providing a more accurate evaluation of this vital signal’s impact on the health of a subject.

**Figure 11:**
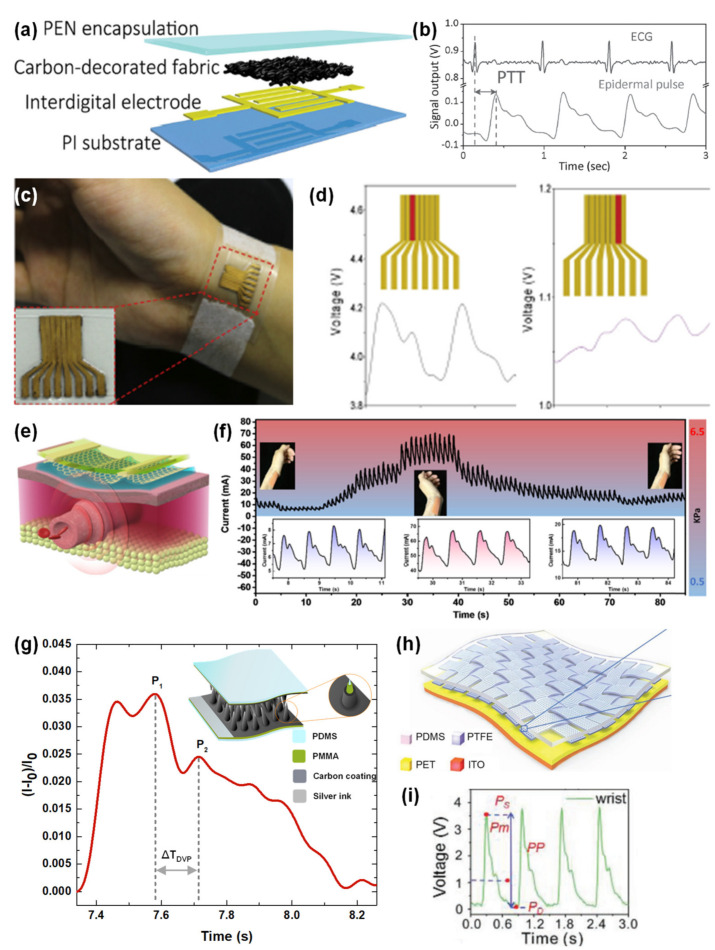
E-skins applied to blood pressure and blood pressure wave detection. (**a**) E-skin developed by Ni Zhao and co-workers in 2016 [32] (Copyright © 2020 WILEY-VCH Verlag GmbH & Co. KGaA, Weinheim, Germany). (**b**) Electrocardiogram signal and epidermal pulse signals from the sensor in (a), with identification of the PTT [32] (Copyright © 2020 WILEY-VCH Verlag GmbH & Co. KGaA, Weinheim, Germany). (**c**) E-skin developed by Yao-Joe Yang and co-workers in 2018, attached to the wrist [181] (© 2020 Elsevier B.V. All rights reserved.). (**d**) Signals measured in the wrist by two elements of the sensor array shown in (c) [181] (© 2020 Elsevier B.V. All rights reserved.). (**e**) E-skin developed by Wei Lu and co-workers in 2019 [182] (© 2020 Elsevier Ltd. All rights reserved.). (**f**) Output of sensor (e) for different wrist positions [182] (© 2020 Elsevier Ltd. All rights reserved.). (**g**) Output of the e-skin (inset) developed by Rui Igreja and co-workers in 2018 when placed over the wrist [22] (Copyright © 2020 WILEY-VCH Verlag GmbH & Co. KGaA, Weinheim, Germany). (**h**) E-skin developed by Zhong Lin Wang and co-workers in 2018 [265] (Copyright © 2020 WILEY-VCH Verlag GmbH & Co. KGaA, Weinheim, Germany). (**i**) Output of the sensor in (h) when worn at the wrist [265] (Copyright © 2020 WILEY-VCH Verlag GmbH & Co. KGaA, Weinheim, Germany).

The BPW continuously acquired at the wrist may be employed in mathematical entities, transfer functions (TFs), to infer systolic and diastolic BP values after a proper wave calibration [260,261]. Due to the recent advances in the acquisition of the radial BPW, especially in the field of e-skin [3,4,5,48,262,263], there is still room for improving this technique and increasing its robustness, simplicity and reliability for practical use, in a continuous way and without limiting the activities of the subject. A radial BPW is composed of an incident wave (generated by blood flow) and two reflected waves (from the hand region and from the lower body) [22,264], as shown in Figure 11g. Its first peak (P_1_) corresponds to the sum of the incident wave and the reflected wave from the hand, while the second peak (P_2_) corresponds to the difference of the reflected wave from the lower body and the end-diastolic pressure [264]. The radial artery augmentation index (A_Ir_), calculated as the ratio between P_2_ and P_1_, is an important indicator of arterial stiffness, as well as the time difference between P_2_ and P_1_ (ΔT_DVP_) [264]. The shape of the wave suffers modifications with aging and hypertension, essentially due to a decrease in blood vessels elasticity [264]. For a proper acquisition of the radial BPW points, it is important that the e-skin sensors present:

- A suitable sensitivity—it should not be too high, to avoid noise amplification [32], yet it needs to be above a certain value to confer the sensor the ability to capture the signal and distinguish its exact shape. Therefore, it should be at least a few kPa^−1^;
- Linearity—over the pressure range involved in the detection of this signal, which is typically below 400 Pa [131] in the absence of an external pressure, to avoid signal distortions [32];
- Fast response and relaxation times—of at least 20 ms for a sampling rate of 50 Hz [32].

Avoiding photolithography techniques for sensor micro-structuring, the group of Ni Zhao developed in 2016 a piezoresistive sensor based on a textile with carbon black particles to specifically detect the BPW at the wrist [32]. Through the pulse transit time (PTT) method, they were able to estimate systolic and diastolic BP values together with electrocardiogram electrodes [32]. Even though this sensor did not present the highest sensitivity ever reported (only 0.088–0.585 kPa^−1^ below 35 kPa), the group successfully proved that higher sensitivities were not beneficial for the BPW detection due to the amplification of noise signals, which deteriorates the signal to noise ratio [32]. Furthermore, the monitoring of the BPW could be enhanced under an external pressure of 19 kPa due to both minimization of the loss of the blood pulses that are perceived by the sensor and higher conformability of the sensor [32]. Nonetheless, higher external pressures would occlude the blood flow and decrease the amplitude of the signal [32]. Figure 11a,b illustrate the developed e-skin and the data acquired for estimation of systolic and diastolic BP values.

The group of Yao-Joe Yang also invested efforts in the design of a piezoresistive sensor that could clearly identify the subtle BPW at the wrist [181]. To achieve such aim, they produced an array of eight sensing elements with a length–width ratio over 8, all aligned with the radial artery length [181]. The sensor also included a micro-structured element of PDMS and MWCNTs with micro-domes produced by demolding from a nylon membrane filter [181]. After placing the sensor over the radial artery, it was possible to guarantee, simply based on the sensor’s simple but clever design, that at least one of the sensing elements could clearly capture the BP [181], as illustrated by Figure 11c,d, where the output of one of the sensing elements clearly captures the BPW, while the other element shows a distorted signal.

Later in 2019, Wei Lu and co-workers employed the already explored technique of using silk as a mold [179] to micro-structure a PDMS film, which was then covered with a self-assembled graphene film and combined with interdigitated electrodes of nickel and gold to form a pressure sensor [182]. The group discovered that the sensitivity of their sensor could be increased by reducing the conducting resistance, which was accomplished by increasing the number of graphene layers over the micro-structures [182]. This would also lead to an increase in the graphene layer thickness, resulting in a larger linear range until a certain value, after which the deformation space between the micro-structures and the electrodes was reduced, thus leading to the opposite effect [182]. Through the optimization of an external pressure applied by a cuff or a medical tape (around 4 kPa), the sensor could be placed at the wrist to detect the BPW even when the subject was moving (walking, cycling, or even running) [182]. Figure 11e,f illustrate this e-skin being worn at the wrist and capable to work with different wrist bending levels.

The e-skin developed by the group of Rui Igreja in 2018, as already mentioned, had a performance capable of detecting the BPW at the wrist [22]. Moreover, this e-skin, that was micro-structured through the laser engraving technique, showed an average response time of only 20 ms, which was enough for the identification of the relevant peaks of the pressure wave, which are employed in the estimation of the A_Ir_ [22], as highlighted in Figure 11g.

One of the most recently reported triboelectric sensors developed by Zhong Lin Wang and colleagues was based on polytetrafluoroethylene (PTFE) strips (micro-structured with nanowires) with an interlaced woven structure over a PET substrate as triboelectric layers, and ITO as electrode, and it was studied for the detection of the BPW at different spots of the human body, especially at the ear and wrist [265]. This sensor was sensitive enough to allow the determination of several relevant cardiovascular parameters, such as the K value, artery compliance (AC), and total peripheral resistance (TPR) [265]. Additionally, by placing two sensors at the ear and wrist, the group was able to estimate the PTT [265]. With the PTT data, they resorted to a linearized model that relates BP with PTT according to Equation (5):

(5) Blood Pressure=a×PTT+b

where *a* and *b* are undetermined coefficients that are specific to each individual [265]. Those coefficients were estimated through a genetic algorithm, and the results were very comparable with those obtained with a standard electronic sphygmomanometer [265]. Figure 11h,i illustrate the e-skin developed by the group, as well as the correspondent output.

#### 3.1.2. Heartbeat

Heartbeat and BP are intrinsically linked, given that both are originated by the heart contraction and relaxation [1]. Heartbeat or heart rate is the number of times the heart beats in a minute, and for a healthy adult, this number is around 60 beats-per-minute (bpm) to 100 bpm at rest [1]. This value is highly variable with age, physical condition, and daily activities, for an adjustment of blood supply to the needs of body tissues, being also affected by health issues [1,266,267]. Bradycardia is the condition in which the heart beats less than 60 bpm, while tachycardia is a heart rate above 100 bpm [1]. Athletes commonly have a larger heart that can pump a greater blood volume with each beat, therefore, their heart does not need to beat as many times to ensure the blood supply—a case of a benign bradycardia [1]. Young children present higher heart rates, reaching an average heartbeat of 145 bpm at the age of 1 month, because their heart is smaller and needs to beat at a higher frequency to supply all the blood needed—a common case of tachycardia [267].

The continuous monitoring of heartbeat may be of significant interest to identify or prevent some health issues related to abnormal bradycardia, tachycardia, or even irregular heart rates. An elevated heartbeat at rest has been shown to correlate with high levels of BP, also being a strong predictor of the development of hypertension and a major risk for coronary heart disease [266]. E-skins capable of monitoring the BP at the wrist can intrinsically measure heartrate. This is possible due to the direct correspondence between one heartbeat and a full BPW cycle. Nonetheless, not all e-skins that can detect the heartbeat are efficient in discerning all the features of the BPW.

**Figure 12:**
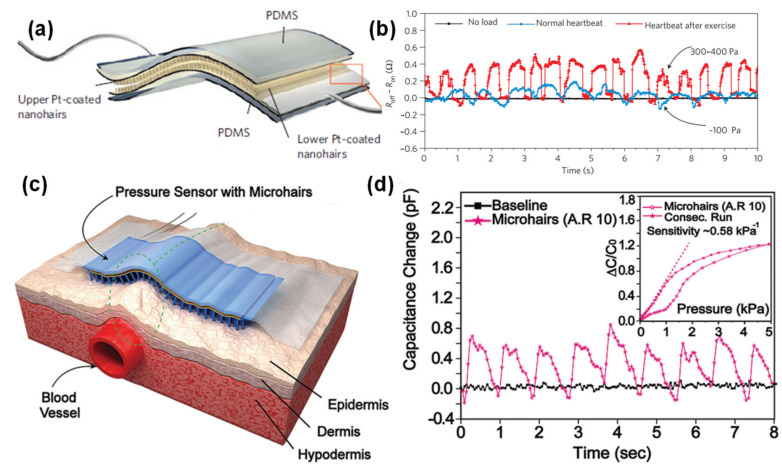
E-skins applied to heartbeat. (**a**) E-skin developed by Kahp-Yang Suh and co-workers in 2012 [131] (Copyright © 2020, Springer Nature). (**b**) Output of the e-skin in (a) during resting or after exercise [131] (Copyright © 2020, Springer Nature). (**c**) Schematic illustration of the e-skin developed by Zhenan Bao and co-workers in 2015 [165] (Copyright © 2020 WILEY-VCH Verlag GmbH & Co. KGaA, Weinheim, Germany). (**d**) Radial artery pulse wave detected by an e-skin in (c), with microhairs with an aspect ratio of 10; the inset shows the sensitivity of the e-skin [165] (Copyright © 2020 WILEY-VCH Verlag GmbH & Co. KGaA, Weinheim, Germany).

Despite being considered a strain sensor, the resistive sensor based on an interlocked array of polyurethane nanohairs covered with platinum, developed by the group of Kahp-Yang Suh in 2012, should be highlighted due to its relevance for the field [131]. This sensor was able to detect pressure, shear stress, as well as torsion. Moreover, when placed on a human wrist, it was capable of detecting the wrist pulse and discriminate between activity states, such as resting (with a maximum signal of 100 Pa) or after exercising (with a maximum signal of 400 Pa) [131], as Figure 12a,b illustrate.

For an accurate detection of the jugular venous pulses or the radial pulse, the group of Zhenan Bao developed a highly conformal capacitive pressure sensor where the key feature was a PDMS film with microhairs that would directly contact the skin, thus increasing the contact area between the sensor and the irregular surface of the skin and consequently amplifying the pulse signal [165]. This sensor could distinguish the jugular venous pulse patterns of healthy subjects and patients with a cardiac disease, ergo promising an alternative to the expensive and complex techniques typically employed in the detection of the jugular venous pulse [165]. Figure 12c,d display the e-skin developed by the group, as well as an example of its output when placed over the wrist.

#### 3.1.3. Respiration Rate

Respiration (or breathing) is an essential biological process by which oxygen is supplied, while carbon dioxide and other metabolic waste products are removed from body tissues [1]. Several structures are involved in respiration, besides lungs. In a normal quiet breathing, the lungs expand and contract through the downward or upward movements of the diaphragm muscle, lengthening or shortening the chest cavity, respectively [1]. For a deeper respiration, abdominal muscles move the ribs up or down, respectively, increasing or decreasing the diameter of the chest cavity [1].

Several factors may affect the respiration rate, such as age [267], activity [1], and illness [268]. While a temporary increase in respiration rate may be an adjustment conducted by the body to provide more oxygen to the tissues in case of an increased demand, as during physical exercise [1], sustained high respiration rates (above 14 to 36 breaths per minute) are important indicators of health issues in several body systems, as well as predictors of adverse health events, which points towards the relevance in assessing this vital sign in a continuous way in critical patients [268].

**Figure 13:**
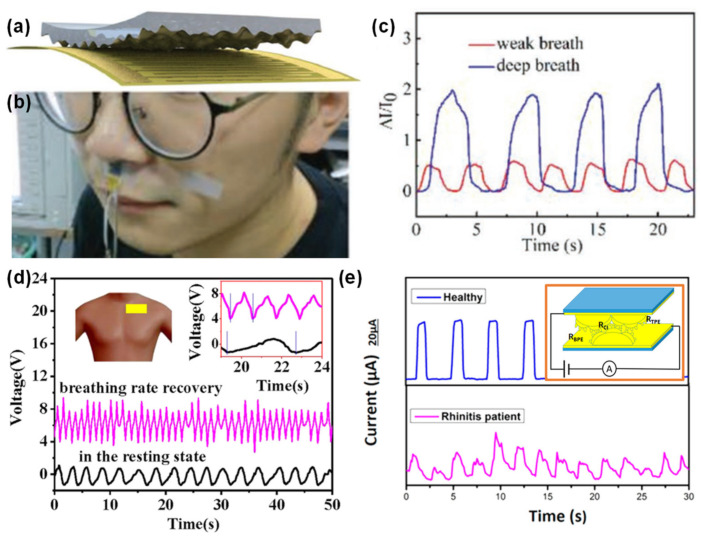
E-skins applied to respiration. (**a**) E-skin developed by Dawen Zeng and co-workers in 2019 [130] (© 2020 WILEY-VCH Verlag GmbH & Co. KGaA, Weinheim, Germany). (**b**) Photograph of the sensor in (a) attached to the skin under the nostrils [130] (© 2020 WILEY-VCH Verlag GmbH & Co. KGaA, Weinheim, Germany). (**c**) Output of the e-skin in (a) when monitoring weak and deep breath [130] (© 2020 WILEY-VCH Verlag GmbH & Co. KGaA, Weinheim, Germany). (**d**) Output of the e-skin developed by Li Wang and co-workers in 2016 to detect respiration changes between rest state (black line) and post-exercise state (purple line) [269] (Copyright © 2020 Elsevier B.V. All rights reserved.). (**e**) Output of the e-skin (inset) developed by Jing Sun and co-workers in 2017 to detect breathing patterns differences between a healthy subject and a rhinitis patient (reprinted with permission from [270]. Copyright 2017 American Chemical Society, Washington, WA, USA).

Many e-skin sensors have been developed and tested for the detection of respiration with potential for disease diagnosis or monitoring. The e-skin developed by the group of Dawen Zeng (described in Section 2.1.3) was tested under the nostrils to detect respiration through air flow, being able to distinguish the pattern differences between a weak breath (less intense and more frequent) and a deep breath (slower yet more intense) [130], as shown in Figure 13a–c.

The group of Li Wang developed a triboelectric sensor based on a rough PET substrate coated with aluminum, in contact with a PTFE film, which could detect the respiration when placed over the chest to monitor the muscles movements [269]. The output signal of this sensor reminds of a triangular wave, whose peaks correspond to chest stretching, while valleys are associated to chest shrinking, with a full cycle being assigned to a single breath [269]. In rest, the sensor detected 19 breaths per minute, while after exercise the number increased to 51 breaths per minute with a more intense output, due to a greater amplitude of muscles movements [269], as displayed in Figure 13d.

Jing Sun and colleagues used a banana leaf as a mold to micro-structure PDMS films with primary, secondary, and tertiary ridges, covered with silver [270]. The e-skin was produced by assembling two films with the micro-structures facing each other, and by placing it under the nostrils, the sensor could detect the air movement induced by respiration [270]. The e-skin was even sensitive enough to distinguish normal breathing patterns from rhinitis patient’ patterns, which are more irregular and weaker as a result of a partial nose block [270], as Figure 13e illustrates. Additionally, this patient presented a faster breathing rate to compensate for the less efficient respiration [270].

#### 3.1.4. Muscles Movements

The muscles movements monitoring can be very useful in a variety of situations, such as speech recognition [144,190,229,271,272], speech therapy [239], or even detection of diseases such as Parkinson disease [144,271] or sleep disorders [271], and may be effectively done by e-skin sensors.

**Figure 14:**
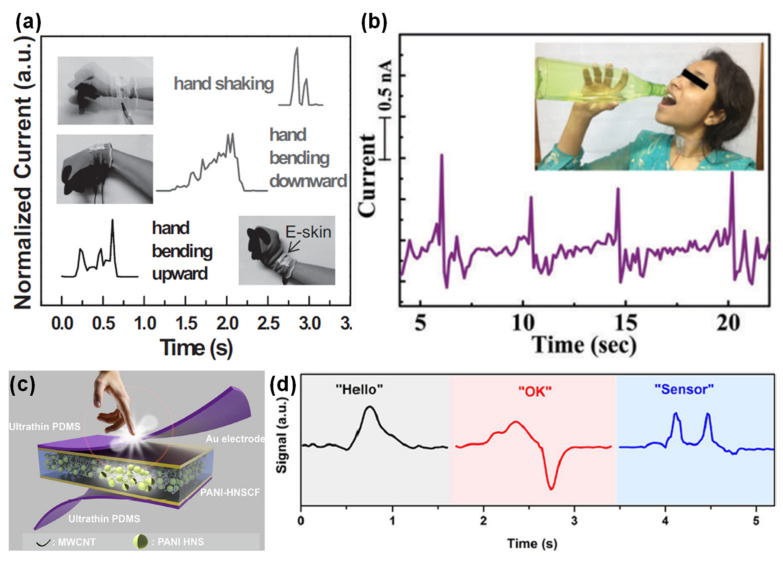
E-skins applied to muscles movements detection. (**a**) E-skin developed by Yang-Fang Chen and co-workers in 2016 for the detection of hand shaking and wrist movement [271] (Copyright © 2020 WILEY-VCH Verlag GmbH & Co. KGaA, Weinheim, Germany). (**b**) Output of the e-skin developed by Dipankar Mandal and co-workers in 2017 when drinking (used with permission of Royal Society of Chemistry, from [272]; permission conveyed through Copyright Clearance Center, Inc.). (**c**) E-skin developed by Guozhen Shen and co-workers in 2017 [239] (© 2020 Elsevier Ltd. All rights reserved.). (**d**) Output of the e-skin in (c) when speaking different words [239] (© 2020 Elsevier Ltd. All rights reserved.).

In 2016, the group of Yang-Fang Chen developed an e-skin composed of a silver nanowires film embedded on the surface of PDMS, suspended above a cloth substrate with conductive threads as bottom electrodes [271]. With extremely high sensitivity values until 3 kPa (over 10^4^ kPa^−1^) and fast response (4 ms) and relaxation times (16 ms), this e-skin was able to consistently detect hand shaking, as shown in Figure 14a, which is extremely useful in the case of patients with Parkinson disease [271]. The detection of tremor by the e-skin could, e.g., trigger a medicine release by another e-skin element to alleviate the symptoms [271]. If mounted on the sleeve opening, the e-skin could also evaluate sleep quality or estimate the walking steps [271]. The e-skin was further mounted in the shirt collar to detect voice patterns with a high consistency, highlighting the potential for conformable electronic throats, which aim at the voice reproduction for patients with vocal chords issues [271].

Dipankar Mandal and co-workers developed a piezoelectric e-skin based on a membrane of electrospun poly(_L_-lactic acid) nanofibers, whose fibers presented a d_33_ of (3 ± 1) pC N^−1^ as a consequence of the electrospinning process, which induces a dipoles alignment perpendicularly to the nanofiber’s length [272]. When attached to the wrist, the e-skin could detect its bending, which is useful for motion monitoring in the context of sports [272]. Furthermore, over the throat, the e-skin could monitor both the movement of the esophagus, distinguishing between drinking [as Figure 14b illustrates] and swallowing, and speech through vocal cords vibration [272]. With a high sensitivity for the differentiation of each letter pronunciation, the e-skin showed a high potential for voice recognition and speech rehabilitation training [272]. Similarly, the e-skin developed by the group of Guozhen Shen, shown in Figure 14c, based on a composite of PVDF, MWCNTs, and polyaniline hollow nanospheres, was placed in the neck to confirm detect muscles movement during speech, being able to distinguish different spoken words [239], as presented in Figure 14d.

The 3-D-printed e-skin developed by Zhengchun Peng and colleagues in 2019 (described in Section 2.1.3) has shown promising results for the monitoring or diagnosis of Parkinson disease due to its fast response and relaxation times (20 ms and 30 ms, respectively), allowing the distinguish of typical vibrations of this disease (5 Hz) [144]. Furthermore, the e-skin was successful in the detection of swallowing, blinking, breathing, and phonation, with very reproducible outputs for the same spoken words, which could be turned into a helpful tool to assist deaf people [144].

#### 3.1.5. Walking and Running

In the context of sports [130] and the diagnosis of health disorders that disturb the gait pattern [168,195], the monitoring of walking or running through an e-skin may provide a valuable contribution.

**Figure 15:**
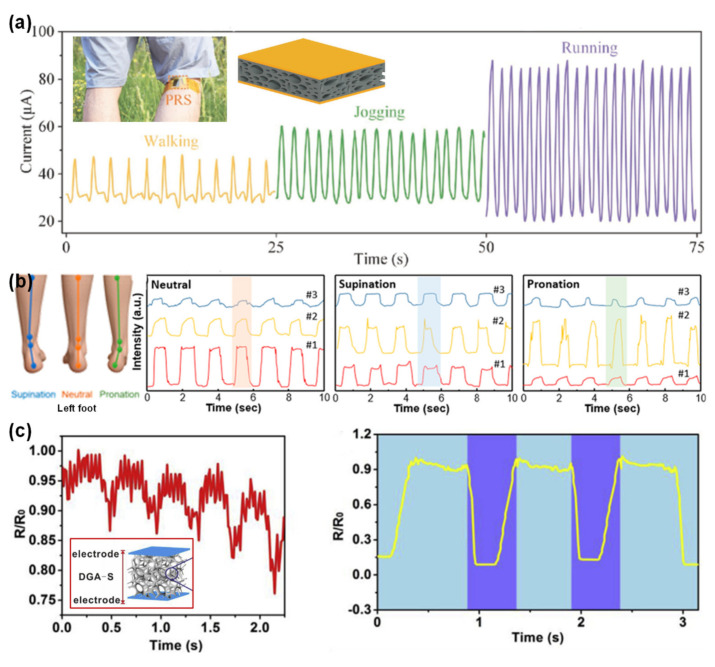
E-skins applied to detection of walking or other related patterns. (**a**) Output of the e-skin developed by Haixia Zhang and co-workers in 2017 for walking, jogging, and running, with the insets showing the placement of the e-skin in the back of the leg and an illustration of the e-skin [232] (© 2020 WILEY-VCH Verlag GmbH & Co. KGaA, Weinheim, Germany). (**b**) Output of the e-skin developed by Tian-Ling Ren and co-workers in 2018 for the discrimination of neutral, supination, and pronation gait patterns (reprinted with permission from [168]. Copyright 2018 American Chemical Society, Washington, WA, USA). (**c**) Output of the e-skin developed by Jing Li and co-workers in 2018 for the detection of the motion caused by the Restless Legs Syndrome (left) or walking (right), with the inset illustrating the e-skin [195] (© 2020 Elsevier Ltd. All rights reserved.).

In 2017, Haixia Zhang and colleagues resorted to the strategy of a sacrificial template (a sugar cube) to produce a sponge of PDMS coated with carbon nanotubes, with electrodes of ITO over PET [232]. With a modest sensitivity (0.03 kPa^−1^) in a large linear range (until 15 kPa), this sensor was attached to the back of the leg to monitor walking, jogging, and running [232]. Due to slight differences between these movements, the muscles of the leg behave accordingly, also presenting some dissimilarities which are successfully captured by the sensor [232], as shown in Figure 15a.

By mimicking the micro-structure of skin through an abrasive papel as mould, the group of Tian-Ling Ren produced an e-skin with two micro-structured PDMS films covered with reduced graphene, with the structures facing inwards [168]. Being capable to detect several stimuli, such as breathing patterns or sound, the e-skin placed in the heel of the foot was able to distinguish between walking, running, and jumping with a high precision [168]. Furthermore, by placing three identical e-skins in specific locations of the foot, it was possible to identify three distinct foot gait patterns, namely supination, neutral, and pronation [168], as illustrated in Figure 15b. This discrimination is possibly due to the distinct concentration forces for each gait pattern, which translate into different output intensities for each e-skin, [168]. This performance has a great potential for the diagnosis of some diseases that affect the human gait, such as neurological disorders, arthrities, or foot deformities [168].

Jing Li and colleagues covered a sea sponge with rGO and silver nanowires to fabricate an e-skin with a sensitivity of 0.016 kPa^−1^ until 40 kPa [195]. Besides the detection of walking, the e-skin could aditionally distinguish subtle legs motion that happens in a sleep disorder called Restless Legs Syndrome, highlighting its diagnosis and monitoring potential [195], as shown in Figure 15c.

### 3.2. Functional Prosthesis and Robotics

With the technological evolution of recent years, many futuristic and even fictional ideas that have been portrayed in cinema are being pursued to become a reality [4]. Nowadays, the prostheses used by amputees are purely cosmetic or employed to help in their movement, lacking a true functionality in the field of sensing, which could be replaced by an e-skin [35,273]. In the robotics field, despite the advantages of soft robots, such as a more robust and safer interaction between robots and humans or the environment [40], or rigid robots with sensing capabilities [144,170], it is necessary to develop stretchable, flexible, and robust electronics that can be conjugated with such creations, a task that is not that trivial. Nevertheless, the e-skin investigation may be the answer to fully potentiate this new generation of robotics.

In the context of robotics and functional prosthesis, instead of presenting impressive sensitivity values, it is more valuable for the e-skins to display a stable sensitivity over a wide range of pressures that are meaningful for the interaction of humans with the surroundings, from less than 10 kPa (linked to gentle touch) to 100 kPa (associated to objects manipulation) [24,41], which contributes to simplifying the signal analysis. This linear range of pressures may be extended to even higher pressures, depending on the purpose of the robots or functional prosthesis, given that foot pressure may exceed 200 kPa [130] and full body weight bearing may reach 1 MPa [36].

**Figure 16:**
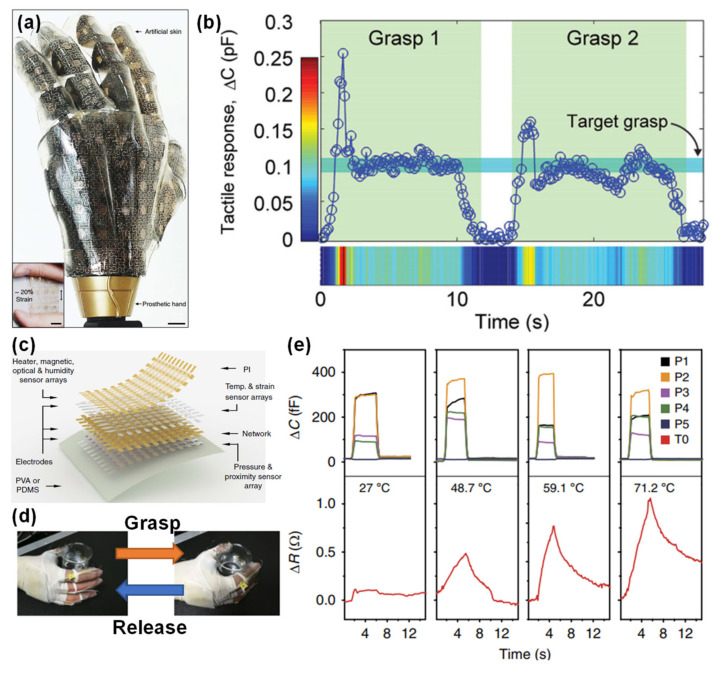
E-skins applied in functional prosthesis. (**a**) Prosthetic hand covered with the e-skin developed by Dae-Hyeong Kim and co-workers in 2014 [35]. The inset shows the e-skin being stretched by 20% (the scale bar is 1 cm) [35] (Copyright © 2020, Springer Nature). (**b**) Output of the e-skin developed by Stéphanie Lacour and co-workers in 2015 for the grasping strength adjustment in real-time [36] (Copyright © 2020 WILEY-VCH Verlag GmbH & Co. KGaA, Weinheim, Germany). (**c**) E-skin developed by Zhong Lin Wang and co-workers in 2018 [273]. (**d**) Photographs of the e-skin in (c) illustrating the grasping and releasing of an object [273]. (**e**) Output of the e-skin in (c) when grasping an object at different temperatures (right). P1–P5 and T0 correspond, respectively, to the different pressure and temperature sensors distributed in the e-skin [273].

In 2014, the group of Dae-Hyeong Kim developed a multi-functional e-skin for functional prosthesis capable of detecting pressure, as well as strain, temperature, and humidity, exploring piezoresistive and capacitive effects [35]. In this e-skin, the sensors comprised ultrathin and flexible single crystalline silicon nanoribbons with linear or serpentine shapes and variable degrees of curvature, allowing a greater sensitivity for mechanical stimuli at the different areas of the prosthesis, and also improved mechanical durability by withstanding larger or smaller degrees of bending [35]. Due to its multi-functionality, a prosthetic hand covered with the e-skin, illustrated in Figure 16a, could monitor the grasping of objects, sense their temperature, and distinguish between dry and wet diapers, which would be an enormous advance compared to regular prosthesis [35]. Furthermore, to approximate the prosthetic hand to a natural hand, the e-skin had thermal actuators to increase the temperature of the prosthesis, mimicking body temperature [35]. To transmit this electrical information to the nervous system, the group also developed a low impedance multi-electrode array through the use of platinum nanowires (to decrease the impedance) and ceria nanoparticles (to suppress an inflammatory response by the body), which was successfully tested in rats [35].

Stéphanie Lacour and colleagues created a stretchable e-skin with capacitive pressure sensors, where the electrodes were stretchable thin gold films and the dielectric layer was a porous silicone foam, and resistive strain sensors with a stretchable thin gold film and elastic liquid metal wires interconnections [36]. When mounted in a glove, the e-skin could provide pressure feedback in real-time, so that the strength of grasping of an object could be quickly adjusted, a crucial achievement in functional prosthesis and robotics [36], as shown in Figure 16b.

In 2018, the group of Zhong Lin Wang created a highly stretchable multifunctional e-skin, shown in Figure 16c, endowed with different sensors to detect pressure, temperature, humidity, strain, light, magnetic field, and proximity [273]. The stretchability was achieved by using meandering wires that connected to each sensor, with a curvature that would reduce with the level of stretching, and each sensor showed low or even null responses to stimuli other than the one they were designed for [273]. This e-skin was highly promising for the functionalization of prosthetic hands capable of sensing pressure, proximity, and temperature [273]. When worn over a prosthetic hand, the e-skin could monitor the grasping strength of an object while sensing its temperature simultaneously [273], as displayed in Figure 16d,e.

**Figure 17:**
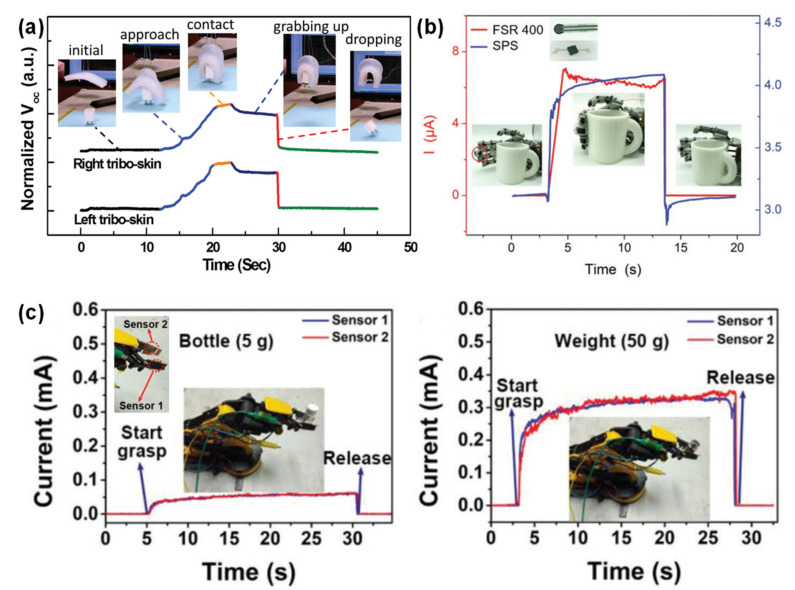
E-skins applied in robotics. (**a**) Output of the e-skin developed by Zhong Lin Wang and co-workers in 2018 for the grasping and dropping of an object by a soft gripper [40] (Copyright © 2020 WILEY-VCH Verlag GmbH & Co. KGaA, Weinheim, Germany). (**b**) Output of the e-skin developed by Zhengchun Peng and co-workers in 2019, worn in a humanoid robotic hand, for the grasping and dropping of an object (SPS), compared to the performance of a commercial sensor (FSR 400) [144] (Copyright © 2020 WILEY-VCH Verlag GmbH & Co. KGaA, Weinheim, Germany). (**c**) Output of the e-skin developed by Vellaisamy Roy in 2019, attached to a robotic arm, when grasping and releasing objects with different weights [170] (Copyright © 2020 WILEY-VCH Verlag GmbH & Co. KGaA, Weinheim, Germany).

In 2018, Zhong Lin Wang and colleagues developed a triboelectric e-skin composed of an ecoflex film with silver flakes as electrode and an ecoflex layer triangular microprisms, integrated into soft robotics as a robotic skin [40]. In a soft gripper it was possible to monitor the approximation to the object, its grabbing, and its sudden drop [40], as Figure 17a illustrates. Due to its irregular surfaces adaptation, the soft gripper could sense the hold and shaking of a doll’s hand [40]. The e-skin could further distinguish wet from dry pants due to triboelectric effect impairment by humidity [40].

With several applications, the e-skin produced by the group of Zhengchun Peng (mentioned in Section 2.1.3 and Section 3.1.4) was placed in the fibers of a humanoid robotic hand, and its performance was compared to a commercial flexible pressure sensor [144]. Besides being able to monitor the grasping and releasing of an object, the developed e-skin could out-perform the commercial sensor concerning the speed at which the pressure changes were detected [144], as shown in Figure 17b.

Besides temperature capabilities, the e-skin developed by Vellaisamy Roy also displayed a great potential for the robotic field when tested in a robotic arm to monitor the pressure change throughout objects grasping and releasing [170]. For the same grasping strength by the robotic arm, the e-skin could distinguish objects by their weight through more intense outputs [170], as Figure 17c shows.

### 3.3. Human-Machine-Interfaces

The development of HMI is of great interest to society, so that some tasks in people’s daily lives may be facilitated, from ludic activities [17,41,274], to moving a wheel chair in an easier and more accessible way [42], or controlling a robot through gestures [275]. An e-skin placed on the human skin or on some objects may open the door to the potentialities of these HMI.

The group of John A. Rogers developed in 2011 an e-skin mimicking a tattoo, endowed with multiple sensors, namely electrophysiological sensors, temperature sensors or strain sensors, as well as several electronic components enabling self-sufficiency [17]. Despite relying on well-established but rigid materials (silicon and gallium arsenide), this e-skin was highly flexible, stretchable (up to 30%), and conformable to the human skin, since the mentioned materials were employed in the form of filamentary serpentine nanoribbons and micro/nano-membranes [17]. This strategy was further adopted in other works [35,90,273]. The tattoo e-skin was tested in a HMI to control a computer strategy game through the different throat muscles movements [17].

**Figure 18:**
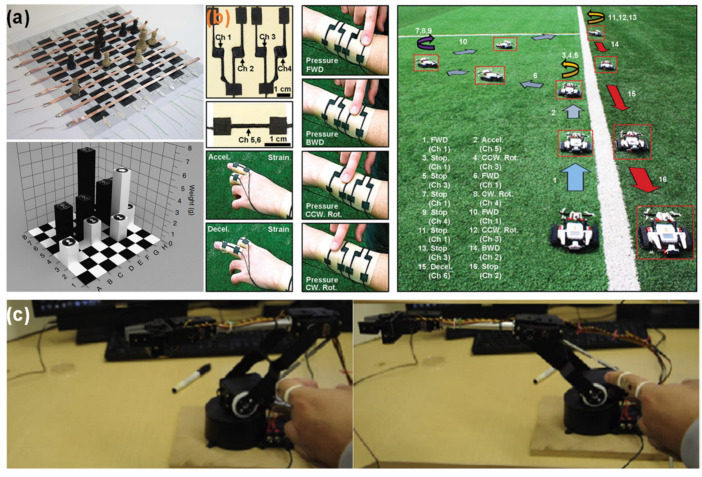
E-skins applied in HMI. (**a**) E-skin array developed by Zhenan Bao and co-workers in 2014 for the detection of chess pieces on a chess board (left), with the respective map reconstruction of the pieces position according to weight (right) [41] (Copyright © 2020, Springer Nature). (**b**) E-skin developed by Dae-Hyeong Kim and co-workers in 2014 for the control of a tank like robot (right), with the identification of the command associated to each pressure or strain sensor (left) [42] (Copyright © 2020 WILEY-VCH Verlag GmbH & Co. KGaA, Weinheim, Germany). (**c**) E-skin developed by Tao Liu and co-workers in 2016 for controlling a robotic arm [275] (Copyright © 2020 Elsevier Ltd. All rights reserved.).

In 2014, Zhenan Bao and colleagues created an e-skin based on a polypyrrole hydrogel (interconnected hollow-sphere structures), micro-structured into triangular lines, which possessed a high sensitivity of 56 kPa^−1^ to 133.1 kPa^−1^ below 30 Pa [41]. With an array of these e-skin sensors placed on a chess board, the group could detect all pieces of the game by assembling specific weights to each piece, according to type and color as Figure 18a displays, demonstrating the potential for HMI [41].

Through an approach based on the formation of reverse micelles (water droplets surrounded by emulsifiers), the group of Dae-Hyeong Kim developed an e-skin with a porous composite of PDMS and MWCNTs that was successfully tested for a HMI [42]. Through the jet printing of the composite on a commercial elastomeric patch, the group create a two-channels strain gauge and a four-channels pressure sensor, where each element was programmed to control one action, from acceleration to deceleration, moving forward or backward, and rotations [42]. This e-skin, worn in the fingers and wrist, was employed in the wireless control of a tank like robot [42], as illustrated in Figure 18b.

In 2016, Tao Liu and colleagues used a laser engraving equipment to produce graphitic structures on a polyimide substrate, in a zigzag pattern, creating an e-skin to be placed over the finger and control the movement of a robotic arm through finger movement [275]. By different finger movements, the robotic arm could move forward or backward, up and down, and grasp or release an object, amongst others [275], as shown in Figure 18c.

In 2018, Zhong Lin Wang and colleagues created a triboelectric e-skin sensor based on a silk fabric, an intermediary layer of carbon nanotubes electrode array, and a nylon fabric [20]. Despite not presenting a high sensitivity value (which varied according to the pressure range, always being lower than 5 × 10^−2^ kPa^−1^), the e-skin was placed on the wrist to control, through finger gestures, the opening or closing of several software in a computer [20]. The e-skin could also control some electrical appliances, such as a light bulb, an electric fan, and a microwave oven [20].

## 4. Conclusions

Although the e-skin field has been growing since the beginning of the XXI century, there is plenty of work left before e-skin devices start spreading in our society and begin to be employed in practical applications. This review highlighted the strengths and fragilities of the micro-structuring strategies commonly employed in the field to improve the performance of pressure sensors. While photolithography techniques are highly precise, their costs prevent the generalized employment for e-skins production to a significant extent. Other low-cost strategies do not offer such a high tailoring of micro-structuring, which prevents fine-tuning in the e-skin performance to adjust it for each application. Some recent strategies, namely 3D printing and laser engraving, may have a high benefit/cost ratio if continued research proves that their precision can be improved and reach the levels of conventional photolithography techniques. Additionally, the raising concern with the sustainability of the planet is inducing researchers to push their efforts towards eco-friendly and abundant materials.

**Figure 19:**
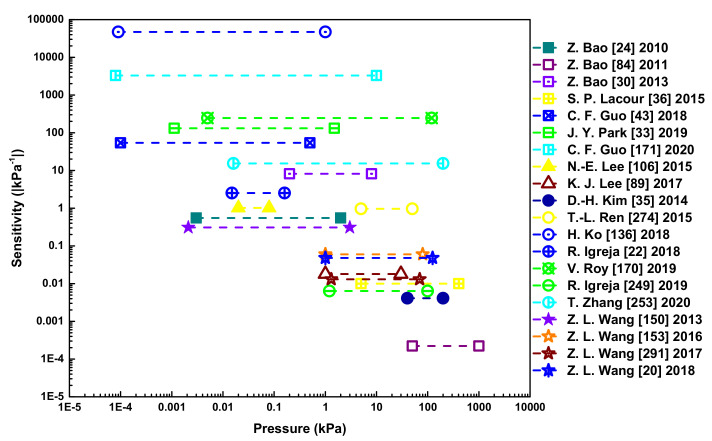
Sensitivity and the corresponding pressure range for which it is valid for 20 of the most cited works since 2010.

The sensitivity and the pressure range for which this parameter is valid is displayed in Figure 19 for 20 of the most cited works since 2010. Despite the absence of a clear trend for the performance of these e-skin pressure sensors, it is visible that the works either reflect a concern about reaching high sensitivities, which might benefit applications that require the acquisition of subtle signals (such as those related to health monitoring), or an attempt to reach a constant, even if more modest, sensitivity over a wider range of pressures, which would be very useful in the context of robotics and functional prosthesis, where the pressures involved may vary greatly from a few kPa until 100 kPa or higher, as previously discussed. By pursuing both high sensitivities and large pressure ranges (as is the case in a few works), these pressure sensors become more versatile since they serve a broader spectrum of applications.

When the needs for both low-cost and tailorable micro-structuring techniques using green materials are simultaneously addressed, the door may open for the generalized society to acquire and start actively using e-skins in their daily lives, either for health monitoring (which has been a trend in the last years) or HMI, robotics, and in the distant future, functional prosthesis, for which the connection between the robotic and the biological parts has still to be deeply investigated and tested before seeing daylight. For this massive usage spread, the multifunctionality of e-skins is also essential, so that they can sense pressure, or other stimuli, while being self-powered and presenting the capability to send data through a wireless communication to other wearables or electronic devices, allowing the user to access the monitoring data in an easy way.

## Figures and Tables

**Table 1:** d_33_ of the most used piezoelectric materials.

Material	BaTiO_3_	PVDF	P(VDF-TrFE)	PZT	ZnO
d_33_ (pC N^−1^)	31.1 * [110]	13–28 [111]	24–38 [111]	593 [112]; 67 * [91]	7.5 * [113]

* Value for a nanowire/nanopillar shape.

**Table 2:** Comparison between the most used transduction mechanisms in pressure sensors [4,5,54].

**Transduction** **Mechanism**	**Advantages**	**Disadvantages**
**Capacitance**	Simple governing equation Simple design and analysis	Power supply required (yet no static power consumption) Limited miniaturization Prone to hysteresis and high response times More complex readout electronics
**Piezoelectricity**	Self-powered Fast response time High sensitivity	Unable to detect static pressure Prone to noise from vibrations or high frequency stimuli Drift in sensor’s response over time Temperature interference Their signal conditioning circuits require power supply
**Piezoresistivity**	Simple structure Simple readout mechanism	Power supply required (with static power consumption) Requires micro-structuring for performance improvement
**Triboelectricity**	Self-powered	Unable to detect static pressure Output affected by frequency of stimulus

**Table 3:** Comparison of the two widely used approaches for the micro-structuring of films, photolithography and unconventional molds, and the emerging laser engraving technique.

**Approach**	**Photolithography**	**Unconventional Molds**	**Laser Engraving**
**Precision**	High	Dependent on the mound	Medium
**Design’s Tailoring**	Possible	Not possible/Highly limited	Possible
**Complexity**	Medium/High	Low	Low
**Time involved**	High	Low	Low
**Costs**	High	Low	Low

## Appendix A

**Table A1:** Key parameters to characterize a pressure sensor [45,54].

Parameter	Description
**Sensitivity**	A measure of the capability of a sensor to transduce a pressure stimulus. It corresponds to the slope of a linear regression to the data plotted as relative output change versus pressure. Sensitivity can be calculated according to: $S=\frac{d\left(\frac{\Delta X}{X_{0}}\right)}{dP}$ where *S* is the sensitivity of the sensor, *X* is the quantitative output signal of the sensor, *P* is the applied pressure, and *X_0_* is the output of the device in the absence of *P*. *X* typically corresponds to resistance (*R*), current (*I*), or capacitance (*C*) measurements.
**Linearity**	The degree to which the performance of a sensor is close to a linear behavior, in a specific pressure range. Given than a sensor is more accurate and reliable in its linear range, the greater the linear range of a sensor the better.
**Limit of Detection**	The smallest pressure that the sensor can distinguish from background noise.
**Hysteresis**	This phenomenon is the incapability of a sensor to return to its original state when the pressure is removed, and it is commonly associated to the viscoelasticity of the materials that compose the sensor. For a pressure sensor it is desirable to have a hysteresis as low as possible so that the performance is reproducible.
**Response Time**	The time spent by the sensor from the instant when it is subjected to a pressure until reaching 90% of a stable output for that pressure, being also negatively affected by the viscoelasticity of the materials.
**Relaxation Time**	The time spent by the sensor to recover its initial state once the stimulus is removed.
**Endurance/Stability**	Evaluated by the number of loading and unloading cycles a sensor may be subjected without significant differences in its output regarding the first solicitations.

## Appendix B

**Table A2:** Features of state-of-the-art pressure sensors based on capacitance (C), piezoelectricity (PE), piezoresistivity (PR), and triboelectricity (TE). “Stability” is the number of loading (L), stretching (S), or bending (B) cycles the sensor was subjected. Symbols: —Breathing; —Functional Prosthesis; —Health Monitoring; —Human Machine Interfaces; —Muscle Movements; —Pressure Mapping; ♡—Pulse wave pressure at the wrist/neck/arm/ankle/head or heart rate; —Robots; —Sound monitoring; —Speech; —Temperature Mapping or Monitoring; —Vibration Monitoring; Health Monitoring; —Walking. Abbreviations and acronyms: Ag—Silver; Al—Aluminum; AR—Aspect Ratio; Au—Gold; B—Bending; CNTs—Carbon Nanotubes; Cu—Copper; elec.—Electrode(s); Electrospin.—Electrospinning; FEP—Fluorinated Ethylene Propylene; IL—Ionic Liquid; ITO—Indium Tin Oxide; L—Loading; LOD—Limit of Detection; MOSFET—Silicon Metal Oxide Semiconductor Field Effect Transistor; MWCNTs—Multi-walled Carbon Nanotubes; Ni—Nickel; NPs—Nanoparticles; NRs—Nanorods; NWs—Nanowires; OFET—Organic Field-Effect Transistor; OTFT—Organic Thin Film Transistor; P(VDF-HFP)—Poly(vinylidene fluorideco-hexafluoropropene); P(VDF-TrFE)—Poly(vinylidenefluoride-co-trifluoroethylene); PANI—Polyaniline; PDMS—Polydimethylsiloxane; PEDOT:PSS—Poly(3,4-ethylenedioxythiophene)–Poly(styrenesulfonate); PEN—Polyethylene Naphthalene; PET—Polyethylene terephthalate; Photolit.—Photolithography; PI—Polyimide; PLLA—Poly(L-lactide); PMMA—Poly(methyl methacrylate); PPy—Polypyrrole; PS—Polystyrene; Pt—Platinum; PTFE—Polytetrafluoroethylene; PU—Polyurethane; PUD—Polyurethane Dispersion; PVA—Polyvinyl Alcohol; PVDF—Poly(vinylidene fluoride); PVP—Poly(4-vinylphenol); PZT—Lead Zirconate Titanate; Rel.—Relaxation; Res.—Response; rGO—reduced Graphene Oxide; S—Stretching; SWCNTs—Single-walled Carbon Nanotubes; Ti—Titanium; TPU—Thermoplastic Urethane Polymer; ZnO—Zinc Oxide.

Type of Sensor	Group, Year	Description	Micro-Structuring Technique	Sensitivity (kPa^−1^), Range of Pressures (kPa)	LOD/Maximum Pressure Tested	Res./Rel. Times (ms)	Stability	Operating Voltage/Energy Consumption	Application
C	Z. Bao [24] 2010	OFET, whose dielectric layer is PDMS with micro-pyramids (6 µm of width).	Photolit.	0.55 (0–2)	3 Pa/ 40 kPa	<300/ <500	10^4^ (L) 15,000 (B)	- 20 V/-	
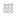
C	Z. Bao [84] 2011	Ecoflex, and elec. of SWCNTs on PDMS.	-	2.23 × 10^−4^ (50–1000)	50 kPa/ 1 MPa	<125/ <125	4 (L) 4 (S)	-/-	
C	Z. Bao [30] 2013	OTFT, whose dielectric layer is PDMS with triangular lines (7 µm of width, 7 µm of height).	Photolit.	8.2 (0–8) 0.38 (8–50)	~ 200 Pa/ 56 kPa	<1/<10	15,000 (L)	- 100 V/1 mW	(♡)
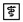
C	D. Zhu [276] 2015	Flexible suspended gate OTFT.	-	192 (0.1–5)	0.3 Pa/ 5 kPa	<10/<10	10^5^ (L)	- 60 V/<1 mW	(♡)
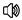
C	Y. Hong [75] 2015	PMMA or PVP, and elec. of PDMS micro-structures (6 µm of height) coated with Ag NWs, and Ag on arylite.	UV/O_3_ treatment	3.8 (0.045–0.5) 0.8 (0.5–2.5) 0.35 (2.5–4.5)	45 Pa/ 10 kPa	<150/ <150	1500 (L) 5000 (B)	0.1 V/-	
C	Z. Bao [165] 2015	PDMS pyramids (6 µm of width, 3 µm of height), PDMS microhairs (30 µm of diameter, AR of 3, 6, and 10), and elec. of PEN and Au.	Photolit.	0.55–0.58 (0–1)	~ 100 Pa/ 10 kPa	-/<30	3000 (L)	1 V/-	(♡)
C	S. P. Lacour [36] 2015	Porous silicone foam with Au elec.	Foam	1 × 10^−2^–1 × 10^−3^ (5–405)	2 kPa/ 405 kPa	7/14	250,000 (L)	-/-	
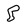
C	S.-D. Lee [72] 2016	Porous PDMS between glass or PET, and ITO elec.	Water mixing and heat	1.18 (0–0.02)	20 Pa/ 5 kPa	150/150	-	-/-	
C	P. Zhu [82] 2017	PVDF dielectric layer, PDMS waves (2.6 µm of width, 800 nm of height), with Ag NWs as elec.	Stretching and plasma treatment	2.94 ± 0.25 (0–2) 0.75 ± 0.06 (2–6.7)	3 Pa/ 6.7 kPa	<50/<50	10^3^ (L) 10^3^ (B)	-/-	()
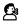
C	C. F. Guo [43] 2018	Ionic gel [P(VDF-HFP) and IL] micro-cones (25 µm of height), with elec. of Ag NWs on PI.	Leaf as mold	54.31 (0–0.5) 30.11 (0.5–10) 8.42 (10–40) 1.03 (40–115)	0.1 Pa/ 115 kPa	29/37	5400 (L)	-/-	()
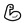
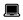
C	C. F. Guo [173] 2018	PDMS micro-towers (6.5 µm of diameter, 14 µm of height) covered with Ag NWs, and Ag NWs on PI.	Lotus leave as mold	1.194 (0–2) 0.077 (2–13)	0.8 Pa/ 13 kPa	36/58	10^5^ (L)	-/-	()
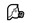
C	Z. L. Wang [273] 2018	Ecoflex, and Ag elec.	-	0.0224 (0–16) 1.25 × 10^−3^ (16–360)	7.3 Pa/ 360 kPa	-/-	-	-/-	
C	C. F. Guo [277] 2018	Rose petal or leaf, or Acacia Mill leaf, with elec. of Ag NWs on PI.	Rose petals and plants leaves	1.54 (0–1) 0.068 (1–40) 0.014 (40–115)	0.6 Pa/ 115 kPa	-/-	5000 (L) 5000 (B)	-/-	()
C	V. Palaniappan [251] 2019	PDMS micro-pyramids, with elec. of Ag on PET, in interlocked design.	Laser engraved mold	2.2 × 10^−3^ (0.075–0.17)	75 Pa/ 170 Pa	-/-	-	-/-	(♡)
C	G. Xing [199] 2019	Polyurethane sponge covered with a composite of silicone rubber, MWCNTs, and graphene nanoplates.	Sponge	0.062 (0–0.3) 0.033 (0.3–4.5)	3 Pa/ 4.5 kPa	45/83	2000 (L)	-/-	(♡ )
C	J. Y. Park [33] 2019	Ionic gel [P(VDF-HFP) and IL] micro-structures (26 µm of size), with elec. of Ag NWs on PDMS.	Sandpaper as mold	131.5 (0–1.5) 11.73 (5–28)	1.12 Pa/ 32 kPa	43/71	7000 (L)	0.5 V/-	(♡)
C	R.-W. Li [86] 2020	Micro-needles (diameter between 166 µm and 422 µm, height between 275 µm and 856 µm) of PDMS and Ni-coated magnetic particles of Ag.	Self-assembly	0.159 (0–1) 0.019 (1.5–11) 4.1 × 10^−3^ (12–145)	1.9 Pa/ 145 kPa	49/51	9200 (L)	-/-	()
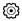
C	C. F. Guo [171] 2020	Iontronic protrusions, and elec. of Au on PI.	Sandpaper as mold	3302.9 (0–10) 671.7 (10–100) 229.9 (100–360)	0.08 Pa/ 360 kPa	9/18	5000 (L) 2000 (B)	-/-	(♡)
C	M. Zhang [278] 2020	Parylene with elec. of Au-coated PDMS micro-pyramids (35 µm of width, 24.7 µm of height) and ITO on PET.	Photolit.	70.6 (0–0.05) 3.3 (0.05–0.325)	1 Pa/ 325 Pa	-/-	10,200 (L)	-/-	(♡)
C	T. Lee [177] 2020	PDMS hierarchical interlocked micro-structures, with elec. of ITO on PET.	Rose petals as mold	0.055 (2–10)	-/10 kPa	300/250	-	1 V/-	(♡)
C or PR	S. Park [85] 2019	Porous PDMS micro-pyramids (50 µm of width), with elec. of ITO on PET (for C sensor), or covered with PPy, with patterned elec. (for PR sensor).	Photolit.	44.5 (0–0.1) (C) 449 (0–0.01) (PR)	0.14 Pa/ 35 kPa (C) or 600 Pa (PR)	50/100 (C) 9/30 (PR)	5000 (L) (C)	1 V/- (C, PR)	(♡)
PE	J. A. Rogers [104] 2013	Aligned fiber of P(VDF-TrFE).	Electrospin.	0.79 V kPa^−1^ (0–0.012) 1.1 V kPa^−1^ (0.4–2)	0.1 Pa/ 2 kPa	-/-	10^3^ (B)	-/-	
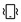
PE	J. A. Rogers [90] 2014	Array of PZT squares connected to the gate elec. of a MOSFET.	-	2 µA Pa^−1^ (0.002–0.01)	0.005 Pa/ 10 Pa	0.1/-	10^3^ (L)	-/-	(♡)
PE	N.-E. Lee [106] 2015	OFET array with P(VDF-TrFE) micro-pyramids (4 µm of width, 2.5 µm of height) as gate dielectric.	Photolit.	1.016 (0.02–0.08) 0.028 (10–100)	20 Pa/ 20 kPa	20/-	10^4^ (B)	-/10 µW	(♡)
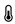
PE	K. J. Lee [89] 2017	PZT thin film on PET, with elec. of Au.	-	0.018 (1–30)	1 kPa/ 60 kPa	60/-	5 000 (L)	-/-	(♡)
PE	Q.-L. Zhao [91] 2017	PZT NWs on planar interdigitated Pt/Ti elec.	-	0.14 V kPa^−1^ (15–70)	15 kPa/ 70 kPa	-/-	10^5^ (L)	-/-	-
PE	D. Mandal [279] 2017	Fish gelatin nanofibers.	Electrospin.	0.8 V kPa^−1^ (0.3–10)	2 Pa/ 25 kPa	16/-	108,000 (L)	-/-	(♡)
PE	D. Mandal [272] 2017	Electrospun PLLA nanofibers.	Electrospin.	3 V kPa^−1^	18 Pa/ 300 kPa	-/-	375,000 (L)	-/-	(♡)
PR	K.-Y. Suh [131] 2012	Interlocked array of PU nanohairs (50 nm of diameter, 1 µm of height) coated with Pt.	Photolit.	-	5 Pa/ 1.5 kPa	50/-	8000 (L)	-/-	(♡)
PR	J.-J. Park [125] 2014	PDMS micro-pyramids (8 µm of width, 4 µm of height) covered with PEDOT:PSS/PUD.	Photolit.	4.88 (0.37–5.9)	23 Pa/ 8 kPa	200/200	800 (S)	0.2 V/-	(♡)
PR	X. Chen [126] 2014	PDMS micro-pyramids (4.5 µm of width) covered with rGO and elec. of ITO on PET.	Photolit.	−5.5 (0–0.1) −0.01 (0.1–1.5)	1.5 Pa/ 1.5 kPa	0.2/-	5000 (L)	1 V/-	-
PR	W. Cheng [38] 2014	PDMS microdomains (18 µm of diameter, 16 µm of height) covered with Au.	Mimosa leaves as mold	50.17 (0–0.07) 1.38 (0.2–1.5) 0.04 (2–10)	10.4 Pa/ 10 kPa	20/-	10^4^ (L) 5000 (B)	0.1 V/-	
PR	W. Cheng [217] 2014	Interdigitated elec. on PDMS sheet with Au NWs coated tissue paper.	Fabric	1.14 (0–5)	13 Pa/ 5 kPa	-/17	50,000 (L) 5000 (B)	1.5 V/30 µW	(♡)
PR	X. Chen [127] 2014	Silicon micropillars (20 µm of diameter, 17 µm of height) covered with Au, and a PPy film on PDMS.	Photolit.	−1.8 (0–0.35)	2 Pa/ 10 kPa	<100/ <100	-	-/-	-
PR	D.-H. Kim [42] 2014	Porous MWCNTs@PDMS with elec. of conductive carbon fabric.	Reverse micelles	-	250 Pa/ 260 kPa	-/-	16 (L)	0.1 V/1 W	
PR	Z. Bao [41] 2014	ITO on PET and Cu foil as elec. and interconnected hollow-sphere structures of PPy hydrogel micro-structured into triangular lines (0.5 mm in height, 1 mm of width).	Photolit.	56–133.1 (0–0.030)	0.8 Pa/ 10 kPa	50/50	8000 (L)	-/-	
PR	D.-H. Kim [35] 2014	Single crystalline silicon nanoribbon with linear or serpentine shapes.	-	4.1 × 10^−3^ (0–200)	-/200 kPa	-/-	-	-/-	
PR	H. Ko [133] 2014	Interlocked array of MWCNTs@PDMS microdomes (4 µm of diameter, 3 µm of height).	Photolit.	−15.1 (0–0.5)	0.23 Pa/ 59 kPa	40/40	10^3^ (L)	10 V/-	()
PR	H. Ko [132] 2014	Interlocked array of MWCNTs@PDMS micropillars (5 µm of diameter, 6 µm of height) with Cu elec.	Photolit.	−22.8 (0–0.05)	10 Pa/ 17 kPa	110/130	-	10 V/-	
PR	T. Zhang [179] 2014	PDMS with micro-structures (11 µm of width, 3.2 µm of height) covered with SWCNTs.	Silk as mold	1.8 (0–0.3)	0.6 Pa/ 1 kPa	10/-	67,500 (L)	2 V/-	(♡)
PR	T.-L. Ren [280] 2015	Carbon black@rubber micro-structures (17 µm of height) with Cu elec.	Abrasive grains as mold	13.8 (0–14.5)	<1 kPa/ 14 kPa	23/-	400 (L)	-/-	-
PR	J. S. Ha [31] 2015	PDMS micropillars (50 µm of diameter, 48 µm of height) covered with Au, and PANI fibers on PET.	Photolit.	2 (0–0.22) 0.87 (0.22–1)	15 Pa/ 9 kPa	50/-	10^4^ (L)	1 V/-	(♡)
PR	T.-L. Ren [274] 2015	Two interlocked films of graphene micro-structures (20 µm of width, 11 µm of height).	DVD laser-scribing	0.96 (0–50) 5 × 10^−3^ (50–113)	-/113 kPa	72/0.4	10^2^ (L)	-/-	
PR	R. Jelinek [193] 2015	Au-coated amine-functionalized PU sponge with Cu elec.	Sponge	−0.31 (0–2) −0.02 (2–10)	10 Pa/ 10 kPa	8/-	10^3^ (L)	-/-	(♡)
PR	K. Cho [139] 2016	PDMS hierarchical micro-domes (59 µm of diameter) with interdigitated graphene elec.	Photolit.	8.5 (0–12)	1 Pa/ 12 kPa	40/30	10^4^ (L)	1 V/-	(♡)
PR	N. Zhao [32] 2016	Nonwoven wood pulp/polyester textile with carbon black particles, and elec. of Au on PI.	Fabric	0.585 (0–35)	-/35 kPa	4/4	5800 (L)	1 mV/3 nW	(♡)
PR	T. Liu [275] 2016	Graphitic structures in PI.	Direct laser writing	-	-/-	-/-	10^4^ (L)	-/-	(♡)
PR	Y.-F. Chen [271] 2016	Ag NWs@PDMS, and conductive threads as bottom elec. in a cloth substrate.	-	1.04 × 10^4^–9.3 × 10^5^ (0–0.1) 2.72 × 10^6^–6.57 × 10^6^ (0.38–3)	0.6 Pa/ 5 kPa	4/16	5000 (L)	0.1 V/-	()
PR	H. Zhang [232] 2017	PDMS sponge covered with CNTs, with elec. of ITO on PET.	Cube sugar as template	0.03 (0–15) 4.8 × 10^−3^ (25–50)	26 Pa/ 150 kPa	300/100	2000 (L)	2 V/-	(♡)
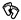
PR	B. Yu [19] 2017	PDMS micro-pyramids (10 µm of width, height) covered with MWCNTs, and elec. of Au/Ni on PET.	Photolit.	−9.95 (0–0.1) −1.5 × 10^−2^ (0.175–0.6)	20 Pa/ 600 Pa	<200/-	20 (L)	1 V/-	-
PR	Y. Lin [129] 2017	PDMS micro-pyramids (11 µm of width, 7.4 µm of height) covered with SWCNTs, and elec. of ITO on PET.	Photolit.	2760 (0–0.4) 8656 (0.4–0.9) 1875 (0.9–1.2)	7.3 Pa/ 1.2 kPa	<4/-	10^4^ (L)	30 V/26.4 nW	(♡)
PR	N.-J. Cho [140] 2017	MWCNTs@PDMS@Sunflower pollen microcapsules, film of PDMS with micro-cubes (150 µm of width), and Cu elec.	Nylon mesh as mold	56.36 (0–1) 2.51 (2–10)	1.6 Pa/ 10 kPa	500/300	25,000 (L)	-/-	(♡)
PR	S. Jeon [161] 2017	PDMS micro-pyramids (20 µm of width) covered with PEDOT:PSS/PUD + Ag NPs.	Photolit.	2.5 (0–0.25)	3 Pa/5 kPa	20/20	10^5^ (L)	0.5 mV/-	(♡)
PR	M. Khine [183] 2017	Interlocked design of PDMS films covered with wrinkled CNT film.	Heat and shrinking	278.5 (0–0.002) 13.2 (0.002–0.025)	0.5 Pa/ 12 kPa	<20/-	500 (L)	1 V/-	(♡)
PR	Z. Tang [189] 2017	Wrinkled graphene films separated by a layer of porous anodic Al oxide.	Chemical synthesis	6.92 (0.3–1.5) 0.14 (1.5–4.5)	300 Pa/ 8 kPa	-/-	10^5^ (L)	1 V/-	-
PR	G. Shen [239] 2017	Film of PANI hollow nanospheres (≈ 414 µm of diameter), MWCNTs, and PVDF, sandwiched between elec. of Au on PDMS.	PS spheres as template	31.6 (0–0.25) 10.61 (1–7)	0.6 Pa/ 50 kPa	100/150	15,000 (L)	1 V/-	(♡)
PR	N. Zhao [281] 2017	Fibers of nylon covered with Cu and doped with carbon black particles and PVDF, with interdigitated elec. of Au on PI.	Nylon fibers	≈ 1 (0–70)	-/60 kPa	2/2	22,000 (L)	1 V/-	(♡)
PR	J. Sun [270] 2017	PDMS mountain (220 µm of width, 30 µm of height) with secondary (35 µm of width, 12 µm of height) and tertiary ridges, and Ag elec.	Banana leave as mold	10 (0–0.4) 3.3 (0.4–1) 0.33 (1–7)	1 Pa/7 kPa	36/30	10^4^ (L)	-/-	(♡)
PR	R. Igreja [22] 2018	PDMS micro-cones (221 µm to 367 µm of diameter, 299 µm to 552 µm of height) covered with carbon coating.	Laser engraving	−2.52 (0–0.16) −0.2 (0.2–1.2) −0.01 (1.6–9)	15 Pa/ 9 kPa	20/-	-	1 V/-	(♡)
PR	T. Zhang [156] 2018	Micro-pyramids (≈ 20 µm of width) of PDMS covered with SWCNTs with a substrate of PEN.	Photolit.	−3.26 (0–0.3) −0.025 (0.6–2.5)	-/2.5 kPa	200/100	5000 (L)	10 V/-	(♡)
PR	H. Ko [136] 2018	MWCNTs@PDMS interlocked micro-domes, micro-pyramids, or micro-pillars (10 µm of diameter/width, 6 µm of height), with Cu elec.	Photolit.	47 × 10^3^ (0–1) 90 × 10^3^ (1–10) 30 × 10^3^ (10–26) (Micro-domes)	0.09 Pa/ 26 kPa	12/12	10^3^ (L)	0.1 V/-	(♡)
PR	W. Yang [39] 2018	Two micro-structured PDMS films covered with Ag NWs in an interlocked design.	Emery paper as mold	9.8 × 10^4^ (0–0.2) 3.5 × 10^3^ (0.2–20)	5 Pa/ 20 kPa	<62.5/ <62.5	10^3^ (L)	0.1 V/1.5 nW	()
PR	J. Yao [190] 2018	ZnO NWs sea-urchin like aggregates, between elec. of ITO on PET.	Chemical synthesis	75–121 (0–0.2) >15 (0.2–10)	0.015 Pa/ 10 kPa	7/9	2000 (L)	5 V/-	(♡)
PR	Y. Gao [205] 2018	Carbonized melamine foam.	Foam	100.29 (0.003–2) 21.22 (2–10)	3 Pa/ 10 kPa	-/-	11,000 (L)	0.1 V/ -	(♡)
PR	S. J. Oh [191] 2018	Conductive Ag nanocrystals on a PDMS film with triangular lines (1 mm of width, 0.5 mm of height), with spacers of insulating Ag nanocrystals.	Self-assembly	2.72 × 10^4^ (0–5) 4.70 × 10^3^ (5–20) 1.09 × 10^2^ (20–100)	10 Pa/ 100 kPa	200/50	200 (L)	0.001 V/ <100 nW	(♡)
PR	X. Peng [229] 2018	Carbon aerogel with cellulose nanocrystals with elec. of Ni on PET.	Freeze-drying	103.5 (0–0.01) 27.5 (0.01–18)	1 Pa/ 18 kPa	-/-	50,000 (L)	1 V/-	(♡)
PR	Y. Fan [282] 2018	Carbonized lignin@PDMS.	-	57 (0–2)	500 Pa/ 130 kPa	60/40	10^5^ (L)	1 V/-	(♡)
PR	S. Shiratori [143] 2018	Two PDMS films micro-structured into fish-scales (0.62 µm of height), covered with PEDOT:PSS and graphene nanosheets, facing each other.	Surface tension	−70.86 (0–1) −1.15 (2–5)	100 Pa/ 5 kPa	82.6/-	-	-/-	(♡)
PR	Y.-J. Yang [181] 2018	MWCNTs@PDMS interlocked micro-domes (3 µm of diameter), with Au elec.	Nylon membrane filter as mold	−6.08 (0–0.15)	-/8.5 kPa	-/-	10^4^ (L)	-/-	(♡)
PR	F. Xuan [37] 2018	Triangular lines of PDMS (10 µm to 14 µm of width, 28 µm to 39 µm of height) covered with CNTs as bottom elec., and smooth PDMS covered with CNTs as top elec.	Laser engraving	−0.11 (0.005–2) −1.28 × 10^−3^ (10–50)	5 Pa/ 50 kPa	200/150	10^4^ (L)	1 V/-	(♡)
PR	L. Li [283] 2018	Pyramid-like structures (4 µm of height) of MWCNTs@PDMS, with an elec. of Au and other of ITO on PET.	Silicon as mold	474 (0–0.4) 14.7 (0.4–1.4) 8.4 (5–110)	0.6 Pa/ 110 kPa	0.002/ 0.074	10^3^ (L)	1 V–5 V/-	(♡)
PR	N. Liu [284] 2018	NWs of PVA sandwiched between interdigitated elec. of Ag/Ni and a wrinkled rGO film.	-	4.52 (0–3) 28.34 (3–10)	2.24 Pa/ 14 kPa	87–155/-	6000 (L)	0.1 V/-	(♡)
PR	L. Li [285] 2018	Micro-pyramids of PDMS covered with PPy, with co-planar Au elec.	Photolit.	1907.2 (0–0.1) 461.5 (0.1–1) 230.1 (1–1.9)	0.08 Pa/ 1.9 kPa	0.05/6.2	15,000 (L)	1 V/-	(♡)
PR	J. Li [195] 2018	Sea sponges with polydopamine, rGO, and Ag NWs.	Sponge	0.016 (0–40)	0.28 Pa/ 40 kPa	-/54	7000 (L)	-/-	()
PR	T.-L. Ren [168] 2018	Micro-structures of PDMS coated with rGO, in interlocked geometry.	Sandpaper as mold	25.1 (0–2.6) 0.45 (10–40)	16 Pa/ 40 kPa	120/80	3000 (L)	-/-	(♡)
PR	R. Igreja [247] 2019	PDMS semi-spheres (320 µm to 340 µm of diameter, 105 µm to 155 µm of height) coated with carbon coating.	Laser engraving	−0.18 (0–0.4) −6.4 × 10^−3^ (1.2–100)	79 Pa/ 100 kPa	-/28	27,500 (L)	5 V–10 V/-	
PR	W. Lu [182] 2019	Micro-structured PDMS covered with graphene, with interdigitated elec. of Ni/Au.	Silk as mold	1875.5 (0–20) 853.2 (20–40)	1.8 Pa/ 40 kPa	2/3	15,000 (L)	1 V/-	(♡)
PR	X. Wang [286] 2019	PDMS with graphene protrusions.	Laser scribing	480 (0–0.1) 34 (0.1–0.4) 0.9 (0.4–1)	28 Pa/ 1 kPa	0.002/ 0.002	4000 (L)	5 V/160 µW	(♡)
PR	D. Zeng [130] 2019	PDMS micro-structures (250 µm, 60 µm, or 15 µm as average height) covered with rGO, with elec. of Au on PI.	Sandpaper as mold	2.5 (0–1) 12 (1–50) 1051 (50–200)470 (200–400)	10 Pa/ 400 kPa	150/40	10^4^ (L)	1 V/-	(♡)
PR	V. Roy [170] 2019	Micro-structured foam (top and bottom sides) of PDMS and graphite, with elec. of ITO on PET.	Sandpaper as mold	245 (0–120) 90 (120–150)	5 Pa/ 150 kPa	8/4	25,000 (L)	-/-	(♡)
PR	Z. Peng [144] 2019	Porous matrix of TPU@NaCl@carbon black particles, with elec. of TPU@Ag particles.	3D printed mold	5.54 (0–10) 0.123 (10–100) 4.8 × 10^−3^ (100–800)	10 Pa/ 800 kPa	20/30	10^4^ (L)	0.3 V/-	(♡)
PR	C. Yang [287] 2019	Au-coated PDMS conical frustum-like structures, with interdigitated elec. of SnSe_2_ nanoplates covered with Au.	Photolit.	433.22 (0–2.4) 2.91 (5–40)	0.82 Pa/ 40 kPa	0.07/0.09	4000 (L)	-/-	(♡)
PR	F. Xuan [250] 2019	PDMS long or short micro-ridges (19.1 µm of width, 20.2 µm of height, 1 mm or 100.5 µm of length), or micro-domes (22.8 µm of diameter, 19.5 µm of height) covered with CNTs.	Laser engraving	−1.82 (0–2) −9.1 × 10^−4^ (10–80)	1 Pa/ 80 kPa	36/52	6000 (L)	-/-	(♡)
PR	W. Huang [212] 2019	Ag interdigitated elec. on cellulose paper, and porous tissue paper coated with Ag NWs.	Tissue paper	1.5 (0.03–30)	30 Pa/ 30 kPa	90/90	-	0.1 V/10 nW	(♡)
PR	Y. Tian [288] 2019	PDMS micro-pillars (500 µm of diameter, 200 µm of height) covered with Ag NWs in interlocked array.	Photolit.	20.08 (0.05–0.8) 3.81 (0.8–2.1)	20 Pa/ 8 kPa	-/-	10^4^ (L)	1 V/-	()
PR	C. Pang [164] 2019	PDMS film with micro-pillars on top (30 µm of diameter, 15 µm or 30 µm of height) and hexagonal structures on bottom (200 µm of width, AR of 1.5), and elec. of graphene on PDMS.	Photolit.	0.015 (0–8) 2 × 10^−4^ (8–20)	20 Pa/ 370 kPa	11/5	10^3^ (L)	-/-	-
PR	B. Zhou [252] 2019	PDMS micro-domes (1000 µm of diameter) with hierarchical pillars (50 µm of diameter), covered with Ag NWs, in interlocked geometry.	Laser cutting and Photolit.	374.5 (0–0.3) 3.86 (0.3–20) 0.63 (20–80)	2.5 Pa/ 80 kPa	-/-	10^4^ (L)	1 V/-	(♡)
PR	P. Liu [244] 2020	NaOH modified tissue paper with silicon rubber, carbon black, and graphene nanoplates as active layer, silicon rubber micro-domes, and interdigitated elec. of Au on PI.	3D printed mold and tissue paper	37.5 (0–2) 2.75 (2–10)	5 Pa/ 10 kPa	50/30	3000 (L)	-/-	(♡)
PR	T. Zhang [253] 2020	PDMS semi-spheres (280 µm of diameter, 200 µm of height) with micro-structures covered with rGO, with interdigitated elec. of Ag NWs.	Laser engraving	15.4 (0–200)	16 Pa/ 300 kPa	15/20	7500 (L)	1 V/-	(♡)
PR & PE	H. Ko [87] 2015	Interlocked array of PDMS micropillars (10 µm of diameter, 10 µm of height) covered with ZnO NWs (coated with Pt or Ni).	Photolit.	−6.8 (0–0.3) −1 × 10^−2^ (0.3–4.6) −6.78 × 10^−5^ (4.6–13.1)	0.6 Pa/ 13 kPa	5/-	10^3^ (L)	10 V/-	
PR & PE	H. Ko [88] 2015	Interlocked array of rGO@PVDF microdomes (10 µm of diameter, 4 µm of height).	Photolit.	-	0.6 Pa/ 49.5 kPa	-/-	5000 (L)	-/-	(♡)
PR & PE	J. Jang [99] 2016	ZnO NRs covered with PVDF, and elec. of rGO.	Chemical synthesis	-	4 Pa/20 Pa	120/-	10^3^ (L)	-/-	(♡)
TE	Z. L. Wang [150] 2013	PDMS film with micro-pyramids (10 µm of width), with an elec. of Au and another elec. of Al with Ag NWs and NPs.	Photolit.	0.31 (0–3) 0.01 (3–12)	2.1 Pa/ 12 kPa	<5/<5	30,000 (L)	-/-	
TE	Z. L. Wang [153] 2016	PET film, a micro-structured PDMS film, and Ag elec.	Dry etching	0.06 (1–80)	1 kPa/ 200 kPa	70/-	10^4^ (L)	-/-	
TE	L. Wang [269] 2016	Rough PET (40 µm of dimension, 0.8 µm of depth) coated with Al, and elec. of Al on PTFE.	Chemical etching	-	-	1/-	-	-/-	(♡)
TE	Z. L. Wang [154] 2017	PDMS film with micro-pyramids (80 µm of width), Ag elec., and SiO_2_ as insulator.	Photolit.	6 × 10^−3^ (0–40)	600 Pa/ 200 kPa	50/-	-	-/-	
TE	S.-W. Kim [289] 2017	Transistor with an ion-gel gate dielectric.	-	0.02 (0–10)	<1 kPa/ 57 kPa	30/-	1700 (L)	0.5 V/180 µW	
TE	Y. Zhang [290] 2017	PDMS film with elec. of carbon fiber.	-	0.055 nA kPa^−1^ (28.2–41.6)	800 Pa/ 41.6 kPa	68/-	10^4^ (L)	-/-	
TE	Z. L. Wang [291] 2017	PDMS or VHB, and hydrogel of polyacrylamide with LiCl as elec.	-	0.013 (1.3–70)	1.3 kPa/ 100 kPa	-/-	20,000 (L) 10^3^ (S)	-/-	
TE	Z. L. Wang [20] 2018	Silk, nylon, and an elec. of CNTs.	Fabric	0.0479 (0–125) 0.0186 (125–350) 0.0033 (350–650)	-/650 kPa	-/-	10^4^ (L)	-/-	
TE	H. Ko [147] 2018	P(VDF-TrFE) and PDMS films with interlocked semi-spheres (100 µm of diameter, 120 µm of height).	Photolit.	0.55 V kPa^−1^ (0–19.8) 0.2 V kPa^−1^ (19.8–98)	-/98 kPa	-/-	10^4^ (L)	-/-	(♡)
TE	Z. L. Wang [40] 2018	Ecoflex with triangular microprisms (1.24 mm of width, 1.64 mm of height), with an elec. of Ag flakes on ecoflex.	Laser grinding machine	0.29 (0–5) 0.005 (5–25)	63 Pa/ 320 kPa	-/-	10^3^ (L)	-/-	(♡)
TE	Z. L. Wang [265] 2018	PTFE strips with NWs (110 nm of diameter, 0.8 µm of height), with interlaced woven structure on PET, and ITO elec.	Plasma dry etching	45.7 V kPa^−1^ (0–0.71) 10.6 V kPa^−1^ (0.71–1.25)	2.5 Pa/ 1.25 kPa	5/-	40,000 (L)	-/-	(♡)
TE	J. Zhou [292] 2018	EVA/Ag film with hollow micro-spheres (750 µm of diameter, 300 µm of height) in both surfaces, with an outer sheet of FEP/Ag.	Hot pressing	18.98 V kPa^−1^ (0.5–1) 0.77 V kPa^−1^ (1.6–10) 0.25 V kPa^−1^ (10–40)	500 Pa/ 40 kPa	-/-	-	-/-	(♡)
TE	Z. L. Wang [176] 2019	Two PDMS films with micro-cones (25.4 µm of diameter, 25.7 µm of height) in interlocked geometry, one covered with Ag NWs and the other covered with PTFE bumps, and a back elec.	Leaf as mold	127.22 V kPa^−1^ (5–50)	5 kPa/ 50 kPa	-/-	5000 (L)	-/-	
TE	X. Chou [293] 2020	PDMS micro-frustum film (14 µm of width, 5 µm of height) covered with Cu with another PDMS micro-frustum film covered with P(VDF-TrFE) in interlocked geometry, and a spacer.	Photolit.	56.7 mV kPa^−1^ (0–600) 15.6 mV kPa^−1^ (700–1000)	-/1 MPa	60/-	80,000 (L)	-/-	(♡)
